# A mathematical model of tumor regression and recurrence after therapeutic oncogene inactivation

**DOI:** 10.1038/s41598-020-78947-2

**Published:** 2021-01-14

**Authors:** Sharon S. Hori, Ling Tong, Srividya Swaminathan, Mariola Liebersbach, Jingjing Wang, Sanjiv S. Gambhir, Dean W. Felsher

**Affiliations:** 1 Department of Radiology, Stanford University School of Medicine, Stanford, CA USA; 2 Molecular Imaging Program at Stanford, Stanford University School of Medicine, Stanford, CA USA; 3 Canary Center at Stanford for Cancer Early Detection, Stanford University School of Medicine, Palo Alto, CA USA; 4 Division of Oncology, Department of Medicine, Stanford University School of Medicine, Stanford, CA USA; 5 Department of Pathology, Stanford University School of Medicine, Stanford, CA USA; 6 Department of Bioengineering, Stanford University School of Medicine, Stanford, CA USA; 7 Department of Materials Science and Engineering, Stanford University, Stanford, CA USA; 8 Present Address: Department of Systems Biology, Beckman Research Institute of the City of Hope, Monrovia, CA USA; 9 Present Address: Department of Oncology, The Second Xiangya Hospital of Central South University, Changsha, People’s Republic of China

**Keywords:** Oncogenes, Computational models

## Abstract

The targeted inactivation of individual oncogenes can elicit regression of cancers through a phenomenon called oncogene addiction. Oncogene addiction is mediated by cell-autonomous and immune-dependent mechanisms. Therapeutic resistance to oncogene inactivation leads to recurrence but can be counteracted by immune surveillance. Predicting the timing of resistance will provide valuable insights in developing effective cancer treatments. To provide a quantitative understanding of cancer response to oncogene inactivation, we developed a new 3-compartment mathematical model of oncogene-driven tumor growth, regression and recurrence, and validated the model using a MYC-driven transgenic mouse model of T-cell acute lymphoblastic leukemia. Our mathematical model uses imaging-based measurements of tumor burden to predict the relative number of drug-sensitive and drug-resistant cancer cells in MYC-dependent states. We show natural killer (NK) cell adoptive therapy can delay cancer recurrence by reducing the net-growth rate of drug-resistant cells. Our studies provide a novel way to evaluate combination therapy for personalized cancer treatment.

## Introduction

Cancers are initiated by genetic changes that occur in oncogenes and tumor suppressor genes. While many genetic changes are required to establish a tumor, the therapeutic inactivation of a single driver oncogene has been shown to induce significant tumor regression in animal models and human cancers. This dependence of cancers on a single oncogene for their survival is termed oncogene addiction^1–4^. Oncogene addiction has led to identification of many therapies that target oncogenes including gefitinib for lung adenocarcinomas, trastuzumab for breast cancers, and imatinib for chronic myelogenous leukemia (CML) and gastrointestinal stromal tumors (GIST)^1^. Despite targeted oncogene inactivation, cancers eventually evolve to develop therapeutic resistance, ultimately resulting in treatment failure and cancer recurrence^5,6^. It remains unclear how cancers evolve to acquire these mutations to become resistant to therapies and whether these mutant cells are pre-existing or only acquired after therapeutic inactivation.

*MYC* is a driver oncogene that is overexpressed in a wide range of human cancers, including hematopoietic, breast, liver, colon, ovarian, lung carcinomas, osteosarcomas, glioblastomas, melanoma and myeloid leukemias^7,8^. *MYC* is a transcription factor that globally regulates gene expression response to cellular growth, proliferation, metabolism, adhesion, survival and DNA repair, as well as more complex programs such as stemness, angiogenesis and the immune response^9–15^. As shown in clinically relevant transgenic mouse models, *MYC* driven cancers are oncogene addicted^3,16,17^.

**Figure 1:**
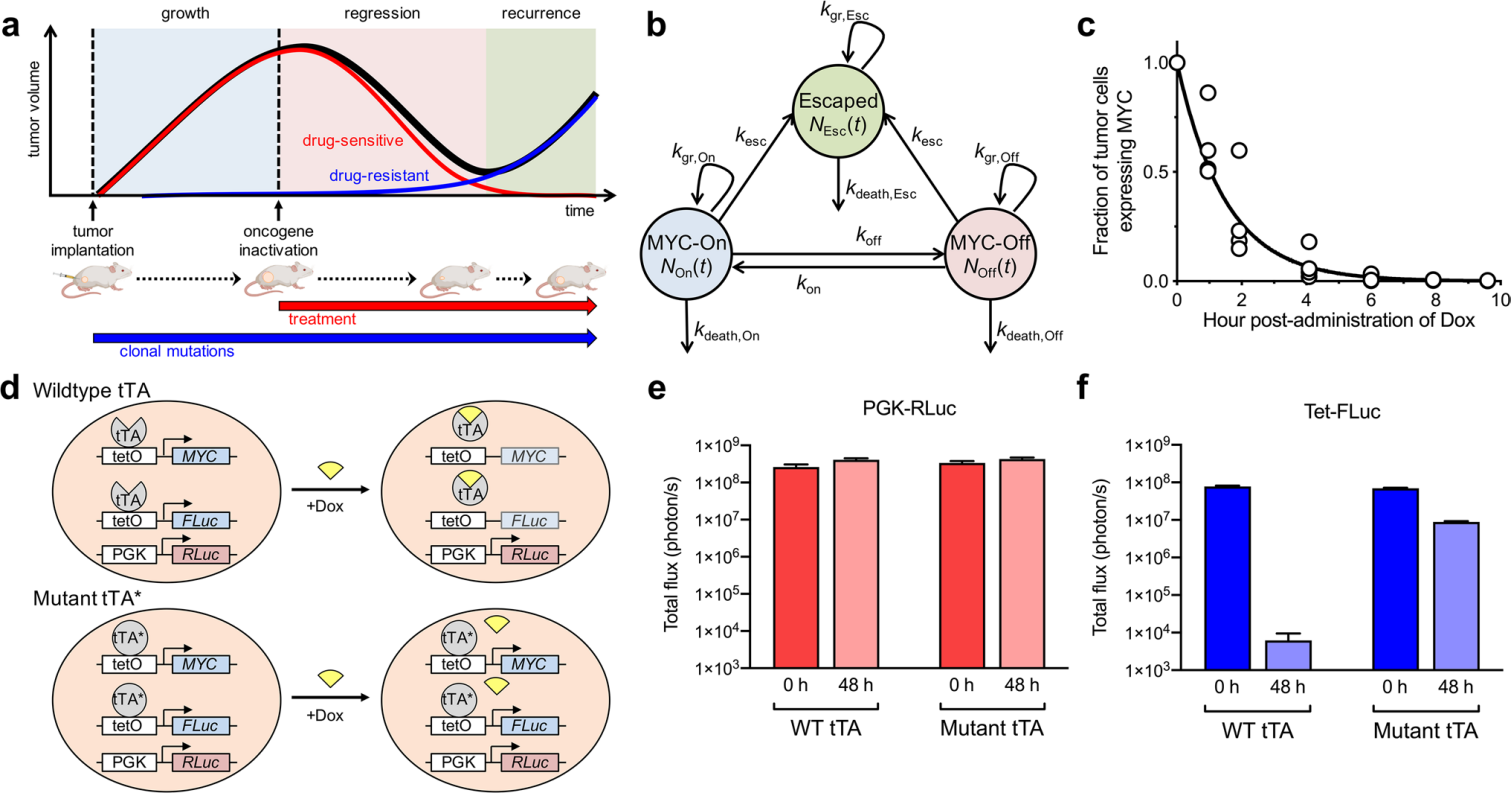
Combined mathematical model and transgenic mouse model to quantify tumor growth, regression and relapse upon oncogene inactivation. (**a**) Kinetics of tumor growth, regression and recurrence following treatment-induced oncogene inactivation. (**b**) Generalizable 3-compartment mathematical model to study drug-sensitive and drug-resistant tumor cell sub-populations following oncogene inactivation. (**c**) Measurement of the kinetics of *MYC* inactivation via in vivo tumor biopsy following doxycycline (Dox) treatment. *MYC* expression was measured by RT-PCR and normalized to the level before treatment. (**d**) Experimental model used to study oncogene addiction and to measure tumor burden and oncogene expression, based on a conditional MYC-driven mouse model using the Tet-system. Cancer cells were virally transduced with a PGK-RLuc reporter and a Tet-inducible FLuc reporter. In wildtype cancer cells treated with Dox, both *MYC* and *FLuc* are inactivated, while *RLuc* remains active. If the cancer cells acquire a mutation in the tTA region where Dox is binding, the cancer cells become resistant to Dox, enabling MYC and FLuc expression to persist in the tumor. Therefore, RLuc signal was used to measure the total tumor burden and FLuc signal provided an indirect measurement of in vivo MYC expression. (**e**–**f**) Examination of RLuc and FLuc reporters in vitro. Luciferase signal was measured in the wildtype cells or in tTA mutant cells in the presence or absence of Dox.

Upon *MYC* inactivation, the majority of tumor cells are eliminated (Fig. 1a). However, *MYC* oncogene addiction involves both tumor-intrinsic pathways including proliferation arrest, apoptosis, and differentiation, as well as tumor-extrinsic pathways including the shut-down of angiogenesis and cellular senescence, which are processes mediated by the host immune system^3,11,16–21^. Thus, in the absence of an intact immune system, *MYC* inactivation fails to elicit complete and sustained tumor regression^18,21^ and eventually a tumor recurs due to the resistance to *MYC* inactivation and the restoration of MYC expression^22^. However, the timing of occurrence of resistance and the frequency of mutation acquisition are difficult to predict during tumor development and treatment.

Mutation acquisition and drug resistance cannot be detected easily using non-invasive methods. Molecular imaging has been widely used in animal models and patients to measure tumor burden and to monitor different biological processes including metabolism, apoptosis, and immune cell trafficking non-invasively^23,24^. However, little information about resistance, including the timing of mutation acquisition and the proportion of drug-sensitive and resistant cells, has been obtained with current imaging approaches. Other methods to detect drug resistance using tissue biopsy are tedious, subject to confounding measures of tumor heterogeneity, and limited by the number of samples. A non-invasive imaging method that provides gross tumor measurements and enables understanding of the mechanisms of drug resistance and tumor recurrence would be valuable.

Mathematical models have been previously used to describe tumor growth, tumor evolution and therapeutic response^25–32^. In the study of cancer drug resistance, for example, stochastic models have been used to determine the likelihood of whether a cancer will have resistant clones at the time of cancer detection^33^ or during the patient’s lifetime^34,35^, but these results are difficult to extrapolate to a specific patient. In contrast, deterministic models have been used to indicate the average number of resistant cells before and after treatment^36^. We have previously shown that quantitative imaging combined with classifier algorithms may be useful for clinically stratifying cancer patients who may benefit from oncogene-targeted therapeutics^25^. We now aim to understand how imaging measurements alone may be used to understand the mechanism of oncogene addiction and the kinetics of tumor regression and recurrence.

Here, we develop a new quantitative, biologically validated mathematical model of tumor regression and recurrence upon oncogene inactivation that utilizes imaging-based tumor burden measurements to characterize the emergence of therapeutic resistance. We validated the predicted number of drug-sensitive and drug-resistant tumor cells in a conditional transgenic mouse model of MYC-driven T-cell acute lymphoblastic leukemia (T-ALL) in which MYC expression can be controlled temporally through the tetracycline-regulated system (Tet system)^3^. By administration of doxycycline, *MYC* is inactivated quickly and the cancers regress. We used our mathematical model to characterize treatment conditions that lead to regression or recurrence in individual immune-compromised mice. We showed that the adoptive transfer of natural killer (NK) cells, and simulated combination treatments varying the death and growth rates of drug-sensitive and drug-resistant populations, may lead to sustained regression of T-ALL. Our study provides an understanding of the mathematical framework underlying therapeutic resistance and the role of the immune system in this process, which can be employed to evaluate new personalized therapeutic approaches for better cancer treatment in the future.

## Results

### Development of a compartmental model of oncogene-dependent cancer dynamics

We developed a 3-compartment model that describes the dynamic processes of cancer growth, regression and recurrence following administration of a targeted therapeutic or treatment (drug) that inactivates a key driver oncogene (Fig. 1b, Table 1). The total cancer burden is composed of cancer cells in three mutually exclusive states: (1) drug-sensitive cancer cells in an oncogene-activated state (On); (2) drug-sensitive cancer cells in an oncogene-inactivated state (Off); and (3) drug-resistant cells in a persistent oncogene-activated state (Esc), regardless of the presence of drug. The total number of cancer cells at a given time *t* is equal to the sum of the number of cells in each state, i.e., *N*_Total_(*t*) = *N*_On_(*t*) + *N*_Off_(*t*) + *N*_Esc_(*t*). For the experimental mouse model studied here, we assumed that the number of cells *N*(*t*) in each state is correlated with optical imaging signal *S*(*t*), i.e., *S*_Total_(*t*) = *S*_On_(*t*) + *S*_Off_(*t*) + *S*_Esc_(*t*).

**Table 1:** Model state variables and parameters.

	Description (units)
**State variable**	
*N*_On_(*t*)	Number of drug-sensitive cancer cells in On state
*N*_Off_(*t*)	Number of drug-sensitive cancer cells in Off state
*N*_Esc_(*t*)	Number of drug-resistant cancer cells in Escaped state
*N*_Total_(*t*)	Number of cancer cells in all states
*S*_On_(*t*)	Optical signal (total flux) of drug-sensitive cancer cells in On state (photon/s)
*S*_Off_(*t*)	Optical signal (total flux) of drug-sensitive cancer cells in Off state (photon/s)
*S*_Esc_(*t*)	Optical signal (total flux) of drug-resistant cancer cells in Escaped state (photon/s)
*S*_Total_(*t*)	Optical signal (total flux) of cancer cells in all states (photon/s)
**Parameter**	
*k*_gr,On_	Growth rate of drug-sensitive cancer cells in On state (day^-1^)
*k*_death,On_	Death rate of drug-sensitive cancer cells in On state (day^-1^)
*k*_netgr,On_	Net-growth rate of drug-sensitive cancer cells in On state (day^-1^)
*k*_gr,Off_	Growth rate of drug-sensitive cancer cells in Off state (day^-1^)
*k*_death,Off_	Death rate of drug-sensitive cancer cells in Off state (day^-1^)
*k*_netdeath,Off_	Net-death rate of drug-sensitive cancer cells in Off state (day^-1^)
*k*_gr,Esc_	Growth rate of drug-resistant cancer cells in Escaped state (day^-1^)
*k*_death,Esc_	Death rate of drug-resistant cancer cells in Escaped state (day^-1^)
*k*_netgr,Esc_	Net-growth rate of drug-resistant cancer cells in Escaped state (day^-1^)
*k*_Esc,On_	Rate of escape of On cells to Escaped state (day^-1^)
*k*_Esc,Off_	Rate of escape of Off cells to Escaped state (day^-1^)
*k*_esc_	Generalized rate of escape of drug-sensitive cells to Escaped state (day^-1^)
*k*_off_	Rate of oncogene inactivation (day^-1^)
*N*_On_(0)	Initial number of drug-sensitive cancer cells in On state
*N*_Off_(0)	Initial number of drug-sensitive cancer cells in Off state
*N*_Esc_(0)	Initial number of drug-resistant cancer cells in Escaped state
*N*_Total_(0)	Initial number of cancer cells in all states
*S*_On_(0)	Initial optical signal (total flux) of drug-sensitive cancer cells in On state (photon/s)
*S*_Off_(0)	Initial optical signal (total flux) of drug-sensitive cancer cells in Off state (photon/s)
*S*_Esc_(0)	Initial optical signal (total flux) of drug-resistant cancer cells in Escaped state (photon/s)
*S*_Total_(0)	Initial optical signal (total flux) of cancer cells in all states (photon/s)

Cancer cells proliferate or die according to state-dependent growth or death rates, *k*_gr,On_, *k*_death,On_, *k*_gr,Off_, *k*_death,Off_, *k*_gr,Esc_ and *k*_death,Esc_. These growth and death rates may be: (a) dependent on oncogene-activation status (e.g., On cells may grow and/or die at the same rate as Esc cells); (b) dependent on drug-sensitivity status (e.g., On cells may grow and/or die at the same rate as Off cells; or (c) independent of cancer state (e.g., each cell state has independent rates of growth and death). Furthermore, these rates may be affected by treatment (drug, radiation, etc.) and/or cancer-immune system interactions.

Only drug-sensitive (On and Off) cells are assumed to be affected by oncogene-targeted therapy. Upon oncogene inactivation, On cells undergo rapid oncogene inactivation to the Off state at rate *k*_off_. Upon removal of treatment, Off cells may revert to the On state at rate *k*_on_^3,19,37,38^. Drug-sensitive cells may acquire genetic mutations at rate *k*_esc_ and become resistant to targeted therapy against the specific oncogene, thereby becoming drug-resistant cells.

The general 3-compartment model consists of a set of ordinary differential equations and is based on the principle of mass balance, in which the change in the number of cells in each state is the summation of the influx of cells into the state minus the efflux of cells from the state. These equations are as follows:

1a $$\frac{{dN}_{\text{On}}(t)}{dt}=\left({k}_{\text{gr,On}}-{k}_{\text{death,On}}-{k}_{\text{esc}}-{k}_{\text{off}}\right){N}_{\text{On}}(t)+{k}_{\text{on}}{N}_{\text{Off}}(t)$$

1b $$\frac{{dN}_{\text{Off}}(t)}{dt}={k}_{\text{off}}{N}_{\text{On}}(t)+\left({k}_{\text{gr,Off}}-{k}_{\text{death,Off}}-{k}_{\text{esc}}-{k}_{\text{on}}\right){N}_{\text{Off}}(t)$$

1c $$\frac{{dN}_{\text{Esc}}(t)}{dt}={k}_{\text{esc}}{N}_{\text{On}}(t)+{k}_{\text{esc}}{N}_{\text{Off}}(t)+\left({k}_{\text{gr,Esc}}-{k}_{\text{death,Esc}}\right){N}_{\text{Esc}}(t)$$

Similar equations may be used if cell state is monitored using optical imaging signal *S*(*t*), which has been shown to correlate with tumor cell number^39^:

1d $$\frac{{dS}_{\text{On}}(t)}{dt}=\left({k}_{\text{gr,On}}-{k}_{\text{death,On}}-{k}_{\text{esc}}-{k}_{\text{off}}\right){S}_{\text{On}}(t)+{k}_{\text{on}}{S}_{\text{Off}}(t)$$

1e $$\frac{{dS}_{\text{Off}}(t)}{dt}={k}_{\text{off}}{S}_{\text{On}}(t)+\left({k}_{\text{gr,Off}}-{k}_{\text{death,Off}}-{k}_{\text{esc}}-{k}_{\text{on}}\right){S}_{\text{Off}}(t)$$

1f $$\frac{{dS}_{\text{Esc}}(t)}{dt}={k}_{\text{esc}}{S}_{\text{On}}(t)+{k}_{\text{esc}}{S}_{\text{Off}}(t)+\left({k}_{\text{gr,Esc}}-{k}_{\text{death,Esc}}\right){S}_{\text{Esc}}(t)$$

All model variables and parameters are summarized in Table 1. We note that this model is generalizable to virtually any driver oncogene and corresponding targeted therapy. The model can be further adapted for other study designs. For example, if a tumor cell population *N*(*t*) with growth rate *k*_gr_ reaches a carrying capacity *K* due to limited resources, a logistic function can be incorporated, i.e., $\frac{dN(t)}{dt}={k}_{\text{gr}}N(t)\left(1-\frac{N(t)}{K}\right)$.

### Application of the 3-compartment model to MYC-driven T-cell acute lymphoblastic leukemia

To determine whether a deterministic approach can account for gross changes in tumor burden before and after oncogene inactivation, we adapted the general 3-compartment model to study the doxycycline-mediated inactivation of *MYC* using Tet-regulated transgenic T-ALL^3^. To initially reduce the complexity of the model parameter space, we developed a subset (submodel) of the general 3-compartment model (Supplementary Fig. 1a) to describe the following in vivo conditions: (1) the host is an immune-deficient NSG mouse lacking T cells, B cells, and NK cells which would otherwise alter growth and death rates of cancer cells; (2) a known number of T-ALL cells is implanted subcutaneously in the flank of the NSG mouse at a given time; (3) the T-ALL cells can be *MYC*-inactivated by continued doxycycline administration via drinking water. Validation of this model in a simplified experimental animal setting is ideal because the number of cancer cells in specific states can be monitored using highly sensitive, in vivo bioluminescence imaging (BLI), in which the luminescence produced is proportional to the number of viable cells^39^. The incorporation of other model features, including immune components and heterogeneous cancer cell types with varying drug sensitivity, can be incorporated later as the appropriate biological data becomes available.

The 3-compartment model for doxycycline-mediated MYC regulation in -ALL is based on the following set of assumptions that correspond to our experimental study:

1. In the absence of doxycycline, the oncogene inactivation rate *k*_off_ is assumed negligible (*k*_off_ = 0), and the cells in the MYC-On state remain activated.
2. In the presence of doxycycline, the oncogene activation rate *k*_on_ is assumed negligible (*k*_on_ = 0), and the cells in the MYC-Off state remain inactivated.
3. The rate of escape, *k*_esc_, is assumed same for both MYC-On and MYC-Off cells, and therefore represents a deterministic (average) rate of escape summarizing the experimental time course being studied.
4. Cells that have acquired mutations enabling resistance are in an irreversible drug-resistant (Esc) state, and all Esc cells have at least one mutation enabling resistance.
5. Initial conditions *N*_Off_(0) = *N*_Esc_(0) = 0 are based on the assumption that all cancer cells are initially in the On state, i.e., *N*_On_(0) > 0. Similarly, *S*_Off_(0) = *S*_Esc_(0) = *S*_0_, where *S*_0_ is the non-zero background optical imaging signal.

To reduce the number of parameters, we further simplified the model to include only the net rates of growth or death in each tumor cell state as follows (Supplementary Fig. 1a):

2a $$k_{{\text{netgr,On}}} = k_{{\text{gr,On}}} - k_{{\text{death,On}}}$$

2b $$k_{{\text{netdeath,Off}}} = k_{{\text{death,Off}}} - k_{{\text{gr,Off}}}$$

2c $$k_{{\text{netgr,Esc}}} = k_{{\text{gr,Esc}}} - k_{{\text{death,Esc}}}$$

such that *MYC*-activated states have a positive net-growth rate (i.e., proliferation exceeds death), and that *MYC*-inactivated states have a positive net-death rate (i.e., death exceeds proliferation). The simplified 3-compartment model (Supplementary Fig. 1a) is the minimal model to account for tumor growth, regression, relapse, as well as the kinetics of *MYC* inactivation and escape, during the time course of doxycycline treatment. This model can further be reduced to other submodels that account for complete regression (Supplementary Fig. 1b,c).

The initial tumor burden represents the total number of tumor cells in all states at any time *t*, i.e., *N*_Total_(*t*) = *N*_On_(*t*) + *N*_Off_(*t*) + *N*_Esc_(*t*), which has been shown to be proportional to BLI signal^39^. We assume that there is an imaging resolution threshold (background signal, *b*) for measuring tumor burden. For clinical imaging, *b* may be the minimum spatial resolution required to distinguish the smallest lesion (e.g., a pixel or voxel). For in vivo animal optical imaging, *b* may be a non-zero photon flux acquired in a healthy mouse. We assumed that cancer BLI signal for each state will only be detectable if it surpasses an optical background threshold *b* = S_0_.

### Measuring rate of MYC inactivation by doxycycline

For the MYC-driven T-ALL, we first measured the rate of oncogene inactivation (*k*_off_), which may vary depending on the treatment and dosage. If the rate of oncogene inactivation is much slower relative to cancer cell proliferation, then it may be possible for the MYC-On cells to continue growing before treatment takes effect. Following intraperitoneal administration of doxycycline, we measured the *MYC* inactivation rate in vivo by acquiring a tissue biopsy of the same tumor at various times and measuring the amount of *MYC* mRNA via qPCR (Fig. 1c). *MYC* mRNA was no longer detectable in 99% of T-ALL cells within 6.7 h of doxycycline administration. We calculated the half-time of *MYC* inactivation (*t*_1/2_ = 1.04 h) and the corresponding inactivation rate (*k*_off_ = (ln 2)/*t*_1/2_ = 16.01 day^-1^), which was significantly faster than the observed cancer cell doubling time (1–2 days). This inactivation rate was assumed to be similar for all mice in our study.

### Bioluminescence imaging to monitor cancer growth and oncogene expression

Cancer cells in all states may be monitored quantitatively using real-time non-invasive reporter gene imaging. We chose to use a conditional MYC-driven T-ALL cell line in which *MYC* is inactivated via the Tet system by administration of doxycycline^3,18^. We tracked T-ALL cells noninvasively by imaging stable expression of the BLI reporter gene Renilla luciferase (RLuc) under the control of a constitutive PGK promoter^40^. In the presence of substrate coelenterazine, PGK-RLuc enables tracking of all T-ALL cells noninvasively in vivo, immediately after subcutaneous cell implantation, and before and after doxycycline-mediated *MYC* inactivation.

To monitor the number of MYC-expressing T-ALL cells, we incorporated a Tet-inducible firefly luciferase reporter (Tet-FLuc, Fig. 1d). Both MYC and FLuc are controlled by the Tet system. Thus, Tet-FLuc reporter enables non-invasive in vivo imaging of MYC expression over time. We generated single-cell clonal populations (clone B11 and E12) of the T-ALL cells dually stably transfected with PGK-RLuc and Tet-FLuc (4188-PGK-RLuc/Tet-FLuc) to enable simultaneous, sensitive monitoring of all T-ALL cells (RLuc) and the MYC-expressing subset (FLuc). We validated the dual-reporter system in vitro and found that while RLuc expression remained high after 48 h of *MYC* inactivation via doxycycline, FLuc expression decreased 8000-fold (Fig. 1e,f). We have previously shown that tumor recurrence results from the emergence of mutations in the tetracycline transactivator (tTA) region of the Tet system^22^. Here, we also showed that the mutant tTA cells become doxycycline-resistant and maintain high expression of both MYC and FLuc (Fig. 1e,f). Therefore, using the Tet-regulated system and dual-luciferase labeled cells, we were able to monitor total tumor burden (RLuc) and MYC expression (FLuc) at any given time by in vivo imaging.

We next examined how the timing of *MYC* inactivation contributed to recurrence or complete regression by administering doxycycline either: (a) when the tumor was already large (0.8 cm^3^); or (b) when the tumor was small (immediately after T-ALL cell injection). For each treatment scenario, we fitted the 3-compartment model to measurements of total tumor volume (RLuc signal) to estimate rates of tumor growth (*k*_netgr,On_, *k*_netgr,Esc_,), death (*k*_netdeath,Off_,), and oncogene escape (*k*_esc_). Each treatment scenario is described below and parameter estimates are provided in the Supplementary Tables 1–10.

### Modeling cancer recurrence after MYC inactivation

**Figure 2:**
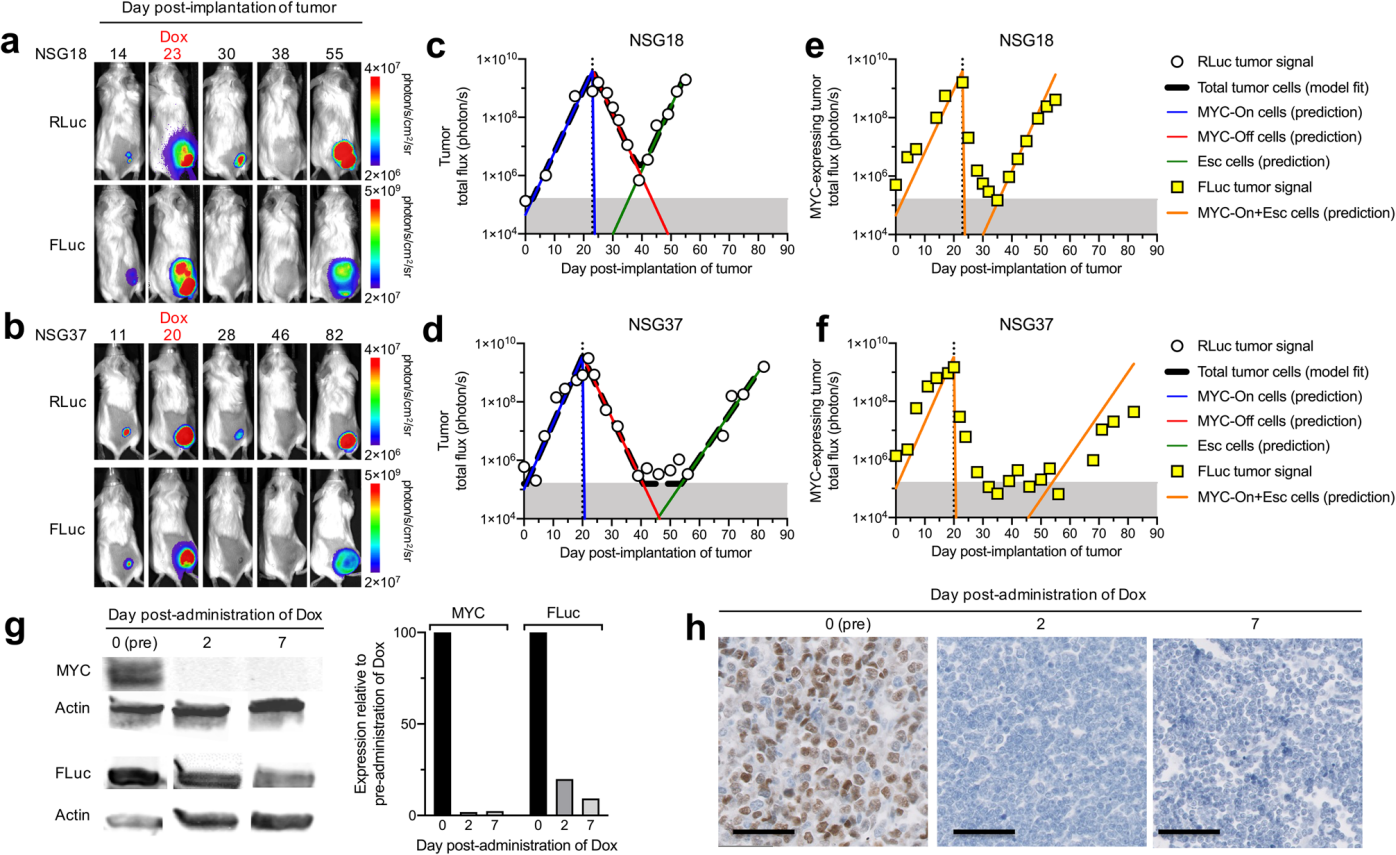
Modeling in vivo bioluminescence imaging (BLI) measurements of tumor burden in mice with recurring tumors. (**a**, **b**) BLI of PGK-RLuc and Tet-FLuc signals in two representative NSG mice treated with doxycycline (Dox) on (**a**) day 23 or (**b**) day 20 after tumor transplantation. (**c**–**f**) Quantitative analysis of the RLuc and FLuc signals from (**a**, **b**). (**c**, **d**) The simplified 3-compartment model was fitted (dashed black line) to RLuc signal (white circles), shown superimposed with the predicted population of MYC-On cells (blue line), MYC-Off cells (red line), and Escaped cells (green line). Shaded grey region (below 1.60 × 10^5^ photon/s) indicates imaging signal is below the limit of detection. (**e**, **f**) The model-predicted MYC-expressing tumor cell population (orange line) superimposed with Tet-FLuc signal (yellow squares). (**g**) Western blot of MYC and FLuc protein expression in the tumors collected before, or 2 or 7 days after *MYC* inactivation. (**h**) Immunohistochemistry staining of MYC in the tumors collected before, or 2 or 7 days after *MYC* inactivation. Scale bar is 50 µm.

**Figure 3:**
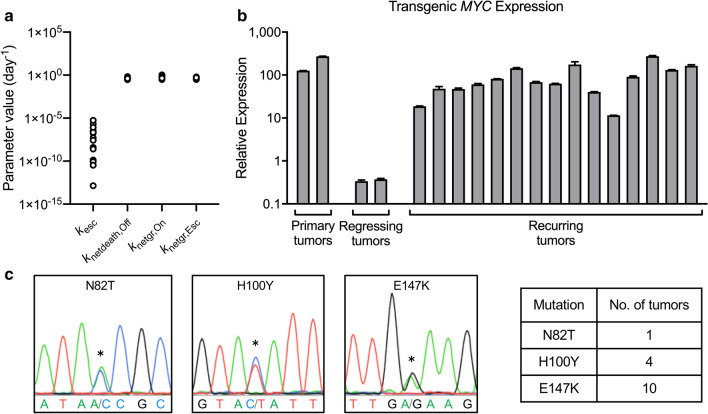
Quantitative analysis of tumor growth, death, and escape rates and associated tTA mutations. (**a**) Summary of parameter estimates from *n* = 15 mice with tumor recurrence: *k*_esc_ (day^−1^), *k*_netdeath,Off_ (day^−1^), *k*_netgr,On_ (day^−1^), *k*_netgr,Esc_ (day^−1^). (**b**) Transgenic *MYC* expression measured by qPCR showed that every recurring tumor exhibited high transgenic *MYC* expression. (**c**) Example chromatograms and table summarizing the N82T, H100Y and E147K mutations detected in the recurring tumors.

When large tumors were treated with doxycycline to inactivate *MYC* (Fig. 2a–h), RLuc measurements showed that tumor initially regressed but ultimately recurred (Fig. 2a,b). The 3-compartment model fitted well to total tumor burden data (Fig. 2c,d) and indicated that tumor net-growth and net-death rates were similar between mice (Fig. 3a and Supplementary Tables). The rate of MYC escape (*k*_esc_), however, differed by several log orders between mice in the same treatment group (e.g., ranging from 3.41 × 10^–11^ to 2.18 × 10^–7^ day^−1^ in Supplementary Table 10), suggesting a mutation event occurring in as few as 20 million cells per day and a diverse mutation frequency even in cancers initiated by a single clonal population of cells in genetically identical subjects (Fig. 3a). We note that *k*_esc_ was estimable with a high degree of uncertainty (112–721% CV, Supplementary Tables  4–7,10), which is difficult to validate non-invasively in the same mouse. The net-growth rates of the doxycycline-resistant population were estimable with reasonable precision (13–53% CV, Supplementary Tables 4–7,10). Therefore, the 3-compartment model was able to characterize which doxycycline-resistant cell populations were fast-growing (e.g., 1.4-day doubling time for NSG18) or slow-growing (e.g., 2.1-day doubling time for NSG37). This suggested that although measurements of gross tumor burden cannot be used to distinguish a specific rate of mutation acquisition for a given mouse, the model is capable of determining which clones are aggressive.

We used the model’s estimated parameter values for each mouse to predict the amount of tumor burden in the doxycycline-sensitive (MYC-On and MYC-Off) and doxycycline-resistant (Escaped) states. As a single clonal cancer population, we assumed all cells were initially in a doxycycline-sensitive MYC-On state (blue curve, Fig. 2c,d, Supplementary Fig. 2). Doxycycline administration quickly converted MYC-On cells to a doxycycline-sensitive MYC-Off state (red curve, Fig. 2c,d, Supplementary Fig. 2). The model predicted that 4188-PGK-RLuc/Tet-FLuc cells acquired resistance to *MYC* inactivation (green curves in Fig. 2c,d and Supplementary Fig. 2) and became imaging-detectable (green curve exceeded the grey shaded region) as early as day 22 in some mice (e.g., NSG17, Supplementary Fig. 2) and as late as day 53 in others (e.g., NSG37, Fig. 2). Furthermore, the model suggested that these Escaped cells could have been detected in these mice as early as day 17 and 45, respectively, if the imaging limit of detection were improved 15-fold (lowered to 1 × 10^4^ photon/s). Therefore, with only a gross BLI measurement of tumor burden, the model can be used to predict the number of drug-sensitive cancer cells at any time. This information is not easily obtained via an invasive biopsy and may be useful for evaluating the efficacy of a MYC-targeted drug.

To validate the 3-compartment model structure, we compared the predicted proportion of cells expressing MYC (orange curve, Fig. 2e,f, Supplementary Fig. 2) to in vivo measurements of MYC expression. For all mice, the model predicted that the MYC-expressing population regressed immediately. However, the FLuc signal lingered for 2–7 days post-administration of doxycycline. We hypothesized that the remaining Tet-FLuc signal was the result of accumulated trace leftover FLuc signal in millions of cancer cells. To confirm this, we measured MYC and FLuc protein levels in excised treated tumors collected before, and at 2 and 7 days after doxycycline administration (Fig. 2g, Supplementary Fig. 3). *MYC* was quickly inactivated, as the model predicted, which is consistent with our earlier in vivo measurements (Fig. 1c), while FLuc expression decreased at a slower rate consistent with our BLI measurements (Fig. 2e,f). The inactivation of *MYC* was further confirmed by immunohistochemical staining of MYC in these excised tumors (Fig. 2h). Therefore, based on our Tet-FLuc measurements and protein expression data, we validated that the model-predicted kinetics of the MYC-expressing cell population were highly reflective of the in vivo cell state.

We next examined the mechanism of cancer recurrence in the transgenic T-ALL mouse model. Our previous work demonstrated that recurrence occurs through the restoration of MYC expression and mutation of the doxycycline-responsive tet-repressor (tetR) domain of tTA^22^. We measured the transgenic MYC expression in all the recurring tumors and sequenced the tTA region. All tumor samples (*n* = 15) showed high expression of transgenic MYC compared to regressing tumors (Fig. 3b) and exhibited at least one of three different mutations (N82T, H100Y, E147K) corresponding to the tetR domain of tTA, consistent with our previous reports^22^ (Fig. 3c).

### Modeling cancer regression after MYC inactivation

**Figure 4:**
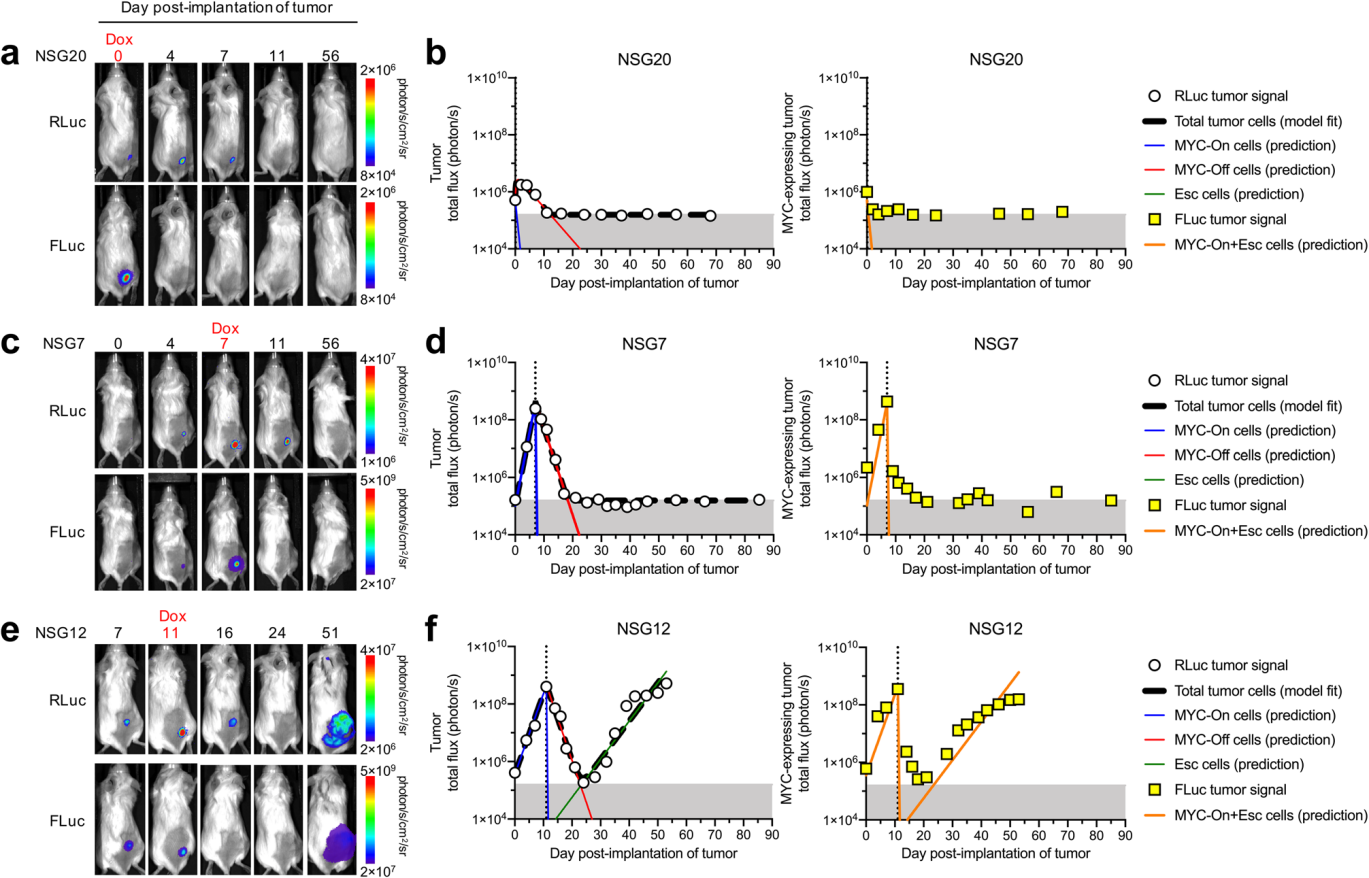
Bioluminescence imaging (BLI) of PGK-RLuc and Tet-FLuc signals in three representative NSG mice treated with doxycycline (Dox) on (**a**, **b**) day 0, (**c**, **d**) day 7, or (**e**, **f**) day 11 after tumor transplantation. (**b**, **d**, **f**) The simplified 3-compartment model was fitted (dashed black line) to RLuc signal (white circles), shown superimposed with the predicted population of MYC-On cells (blue line), MYC-Off cells (red line), and Escaped cells (green line). Shaded grey region (below 1.60 × 10^5^ photon/s) indicates imaging signal is below the limit of detection. The model-predicted MYC-expressing tumor cell population (orange line) is shown superimposed with Tet-FLuc signal (yellow squares).

We hypothesized that earlier treatment of the cancers prior to mutation acquisition would increase the likelihood of complete regression. To test this, we inactivated *MYC* in separate sets of mice at various times post-implantation of cancer cells and used subsets of the 3-compartment model (Supplementary Fig. 1b,c) to describe each mouse’s RLuc measurements of total tumor burden (Fig. 4 and Supplementary Figs. 4, 5). We found that when cancers were treated on the day of implantation or up to 7 days post-implantation, all cancers regressed completely (Fig. 4a–d and Supplementary Figs. 4, 5). We observed when treatment was administered as late as 11 days post-implantation of cells, 2 of 3 cancers relapsed (Fig. 4e,f and Supplementary Figs. 4, 5). This suggests that for the T-ALL 4188-PGK-RLuc/Tet-FLuc cells, complete regression may be best achieved when treatment is initiated during the first week of cancer growth.

**Figure 5:**
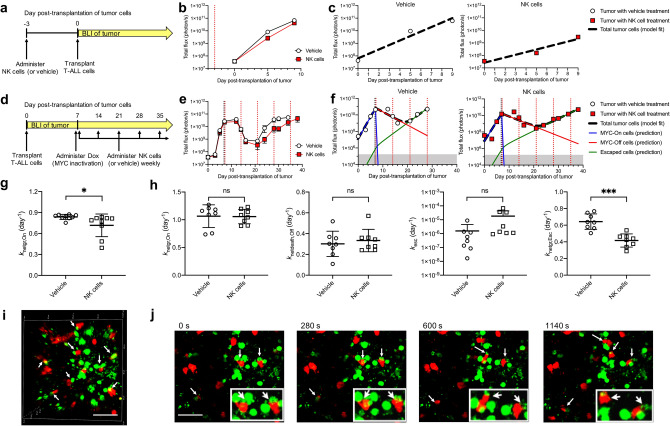
Bioluminescence imaging (BLI) and mathematical modeling analysis showed NK cells delayed (**a**–**c**) tumor growth and (**d**–**f**) recurrence. (**a**) NK cells were adoptively transferred 3 days before intravenous T-ALL-FLuc cell transplantation. (**b**) FLuc measurement of tumor burden in mice with or without NK cell transfer (mean ± SEM of *n* = 9 mice per group). (**c**) Representative growth curve for a mouse without or with NK cell transfer. (**d**) T-ALL-FLuc cells were intravenously transplanted into NSG mice on day 0. On day 7, doxycycline (Dox) was administered. Either NK cells or a saline control were transferred on day 7 and weekly thereafter. (**e**) BLI measurement of tumor burden in mice with or without NK cell transfer (mean ± SEM of *n* = 8 mice per group). (**f**) Examples of model fits to BLI measurements in representative mice without or with NK cell transfer. The simplified 3-compartment model was fitted (dashed black line) to FLuc data (white circles), shown superimposed with the predicted population of MYC-On cells (blue line), MYC-Off cells (red line), and Escaped cells (green line). Shaded grey region (below 1.60 × 10^5^ photon/s) indicates imaging signal is below the limit of detection. (**g**) Comparison of *k*_net,growth,On_ in mice shown in (**a**–**c**). *p < 0.05. (**h**) Comparison of the parameter estimates for mice shown in (**d**–**f**). ***p < 0.0001. (**i**–**j**) Intravital microscopic imaging showed NK cells (mCherry-labeled, red) attacking T-ALL cells (GFP-labeled, green). (**i**) 3D imaging of interactions of NK cells and tumor cells. (**j**) Time-lapse imaging showed NK cells (red) actively attacking T-ALL cells (green). Zoomed-in images are shown n inset. Interactions of NK cells and tumor cells are indicated by white arrows. Scale bar = 20 µm.

### Model-predicted therapeutic effect of natural killer (NK) cells

The 3-compartment model serves as a quantitative framework to study how immune cells may alter the kinetics of tumor growth and death. We applied our mathematical model to study how NK cells affect cancer progression and treatment (Fig. 5). We have recently shown that NK cells can alter the fate of MYC-driven cancers^41^. Here, we first examined the net growth and death rates of cancer cells in the presence and absence of NK cells in immune-deficient NSG mice (Fig. 5a, Supplementary Fig. 6). Compared to the mice without NK cell transfer, the mice receiving syngeneic NK cell (CD3^−^ NKp46^+^) adoptive transfer 3 days before T-ALL transplantation showed significantly slower cancer growth (0.72 vs. 0.84 day^−1^, p < 0.04 using an unpaired t-test, Fig. 5b,c,g). We further validated this using intravital microscopic (IVM) imaging. We visualized NK cells (red, derived from mCherry transgenic mice) infiltrating in the tumor microenvironment and actively attacking cancer cells (green, labeled with PGK-GFP) in real time in live mice (Fig. 5i–j, Supplementary Videos 1–2).

**Figure 6:**
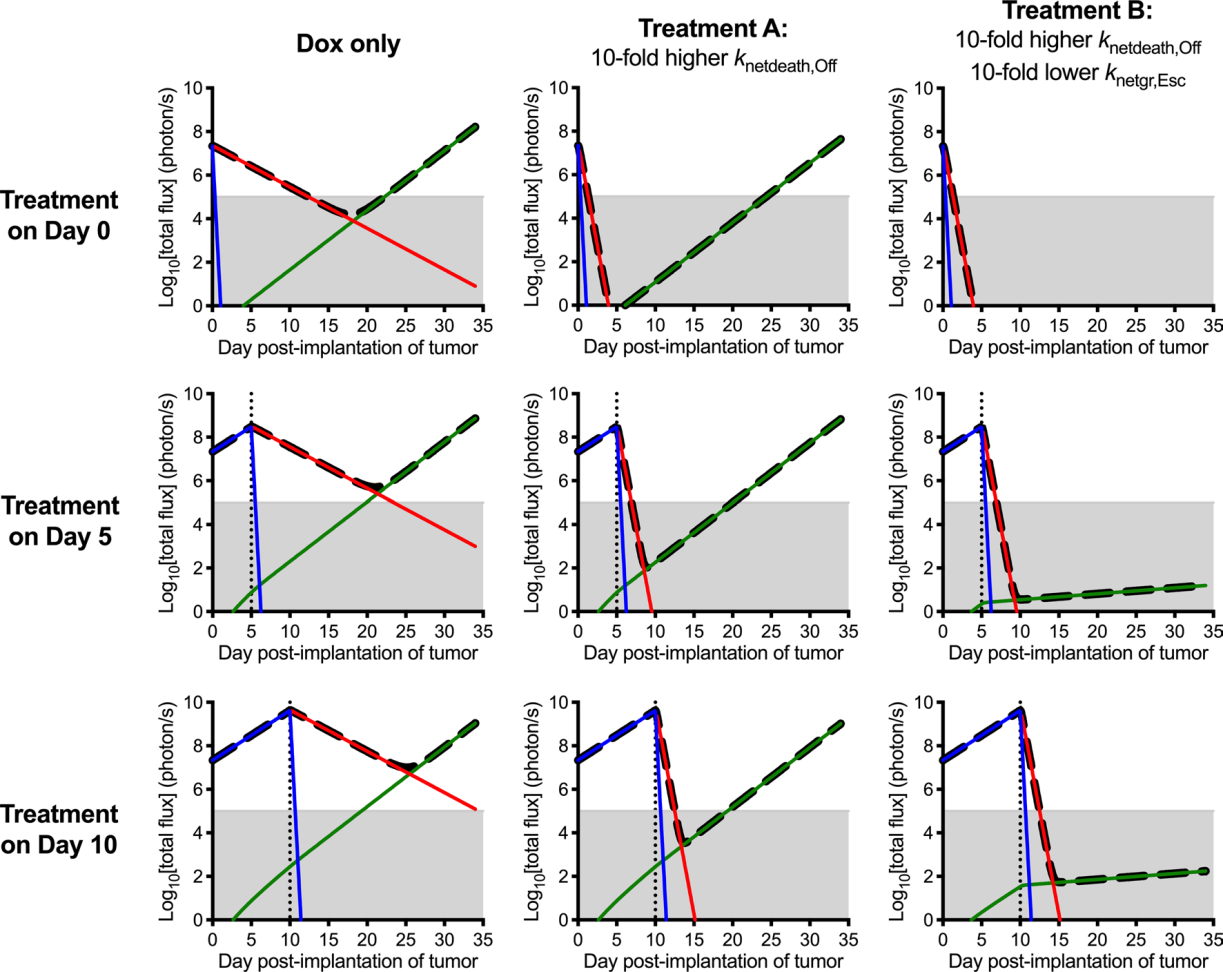
Comparison of doxycycline (Dox) treatment to two hypothetical treatments for a simulated lesion of 1 × 10^7^ T-ALL cells. Shown are the MYC-On (blue), MYC-Off (red), and Escaped (green) cell populations following a one-time treatment dosing on the indicated day. Shaded grey region indicates tumor burden not detectable with imaging. Treatment A has a tenfold higher *k*_netdeath,Off_ relative to Dox, and Treatment B has a tenfold higher *k*_netdeath,Off_ and tenfold lower *k*_netgr,Esc_ relative to Dox.

We then used the 3-compartment model to examine the potential changes in net-growth and net-death rates of the cancer cells following repeated weekly NK-cell adoptive transfer during cancer regression and recurrence (Fig. 5d–f, Supplementary Fig. 7). In this experiment, NK cells were adoptively transferred on the day of *MYC* inactivation and then weekly thereafter. As measured via BLI, cancer recurrence was delayed in the mice that received NK cell transfer compared to mice receiving a saline control. We found that mice receiving NK-cell adoptive transfer showed slower post-treatment net growth rates (0.42 vs. 0.64 day^−1^, p < 0.0001 using an unpaired t-test, Fig. 5h). These results demonstrated that NK cell-based therapy may be particularly effective against MYC-driven lymphoma. NK cell adoptive therapy after *MYC* inactivation did not significantly impact the pre-treatment net growth (*k*_netgr,On_), post-treatment net death (*k*_netdeath,Off_), or escape (*k*_esc_) rates, suggesting that *k*_netdeath,Off_ is primarily controlled by autonomous processes such as apoptosis and proliferative arrest, and *k*_esc_ is independent of NK cell interactions (Fig. 5h).

### Model-based simulations to sustain cancer regression using oncogene-targeted therapeutics

We next used the 3-compartment model and mean values of the optimized parameter estimates (Fig. 3a) to simulate later-stage (50-fold larger) cancers and the potential effectiveness of other therapeutic strategies (Fig. 6). These strategies include starting treatment at an earlier time and leveraging treatments that decrease the growth rate and increase the death rate of drug-sensitive cancer cells. The model indicated that sustained cancer regression is difficult to achieve in the absence of the immune system ven if *MYC* is inactivated arly(e.g., at diagnosis). In contrast, a hypothetical treatment (Treatment A) that increases the death rate of drug-sensitive cells by tenfold may prolong the emergence of a resistant cell population, but the model suggests that even then the cancer eventually recurs. Ultimately a single drug or panel of drugs (Treatment B) that utilizes a combined approach of increasing drug-sensitive cell death and decreasing drug-resistant growth would effectively prolong recurrence. These examples illustrate the potential ability of a biologically validated mathematical model to simulate the effects of timing and combinations of treatments (chemotherapy, adoptive cell therapy) to optimize sustained regression. Therefore, the model may help quantitatively evaluate the potential role of candidate therapies and immune effectors to target MYC and other oncogenes during the process of cancer growth and regression.

## Discussion

We established and validated a new 3-compartment model that quantifies the expression levels of a driver oncogene such as *MYC* in individuals with an oncogene-addicted cancer. The model used imaging-based measurements of tumor burden, which can be commonly obtained in the clinic, to quantify the kinetics of cancer growth, regression, and recurrence in various treatment stages of an individual’s cancer, and to characterize the emergence and progression of resistant clones. The mathematical model can quantify the effectiveness of novel drugs or treatments such as NK cell adoptive transfer, which we showed delayed cancer recurrence by slowing down the net-growth rate of resistant tumor cells. Our approach can aid in the design and evaluation of optimal treatment regimens involving single or combined oncogene-targeted strategies with the incorporation of immune-based therapies.

Our mathematical model quantitatively links the mechanism of oncogene inactivation to cancer response in a transgenic MYC-driven cancer. We have reported that oncogene addiction involves both autonomous and immune-dependent mechanisms^3,11,16–21^. Cancers recurred due to mutation and restoration of MYC in the immune-deficient host^22^. Our mathematical model showed that even cancers derived from a single clonal cell in genetically identical hosts can acquire therapeutic resistance at drastically different rates, suggesting that mutation acquisition occurs stochastically among individuals. The kinetics of cancer regression were highly dependent on cancer intrinsic changes, but the clearance of the resistant cell population likely requires the immune system^18,21^. We quantified the rate at which NK cells eliminate treatment-resistant cancer cells, which may help optimize NK-cell therapy to further reduce cancer recurrence (Fig. 5). Other immune components such as CD4^+^ T cells are also required to eradicate cancer recurrence by different mechanisms^18,21^. This suggests that combination targeted and immune therapies may best reduce cancer recurrence, and our mathematical model provides a new way to analyze these outcomes quantitatively for each therapy.

The 3-compartment model is the minimal model needed to describe the mechanisms of oncogene inactivation and mutation acquisition and provides a fundamental quantitative starting point to study how the immune system may affect the success of a cancer treatment. We will focus next on incorporating additional compartments into the minimal model to account for regulatory effects of the immune system, including NK cells, CD4^+^ and CD8^+^ T-cells, and macrophages^18,20,21,41^. We are developing new imaging techniques to track different immune populations such as activated T cells and NK cells in vivo^42–46^. Novel and specific imaging probes can now visualize and quantify cellular changes including proliferation^47^, apoptosis^48^ and senescence^49^. The development of more sophisticated mathematical models is becoming possible with advancements in functional and anatomical imaging modalities that boast improved temporal and spatial resolution^23,24^. These mathematical models may eventually describe the spatial heterogeneity of the tumor microenvironment and include stochastic cellular processes that are critical for small tumors (birth, death, and clonal evolution of cells).

The predictive capability of any computational model is limited to the data at hand but can be refined using more sensitive imaging and detection methods that boast higher temporal and spatial resolution. For example, in this study, we used BLI to measure disease burden of a mouse model of T-ALL. The development of advanced biomedical imaging techniques to monitor immune cell trafficking will enable further biological and kinetic studies of tumor regression and recurrence in animals and patients. Machine learning-based approaches are now becoming capable of predicting patient relapse at the time of diagnosis^50^, by leveraging extensive data sets involving thousands of measurements to train and validate the model. As state-of-the-art genomic and proteomic methods evolve in the decades to come, thereby enabling the detection and monitoring of early mutations in cancer, we envision that mechanistic modeling and artificial intelligence-based approaches may be integrated to understand, improve and predict the outcome of personalized medicine approaches.

We utilized experimentally validated biological hypotheses underlying oncogene addiction to develop a general mechanistic model that may now aid in the design, evaluation, and analysis of personalized therapeutics in many ways. First, our model can be adapted for other driver oncogenes, oncogene-addicted cancers, and targeted therapeutics. Second, our model can simulate potential treatment options or combinations of treatments including immune therapy, to identify the optimal timing and dosage for a given individual, based on experimentally validated and mechanistically modeled concepts of oncogene addiction. These treatments may include new targeted therapies, alone or in combination with adoptive immunotherapy, and will be critical for establishing the success of personalized medicine.

## Methods

### Cloning and cell culture

The conditional murine T-ALL cell line 4188 was derived from *SRα-tTA/Tet-O-MYC* mice. The cells were cultured in RPMI 1640 medium (Invitrogen) supplemented with 10% fetal bovine serum, 1% Anti-Anti and 50 µM 2-mercaptoethanol (Sigma-Aldrich). *MYC* was inactivated with doxycycline (20 ng/ml, Sigma-Aldrich, T7660).

The RLuc sequence was cloned into a pMSCV retroviral vector (Clontech, CA). The Tet-inducible luciferase reporter (Tet-3G-Fluc) was purchased from Clontech. Virus production and infection were performed as previously described^17^. Retrovirus was produced using the Phoenix retroviral packaging system (ATCC, VA). Lentivirus was produced using HEK293T cells (ATCC, VA). Leukemia cells were spin infected and selected with geneticin (400 µg/ml) and puromycin (2 µg/ml). Two single cell clones (B11 and E12) were finally selected for further in vivo experiments.

### Tumor transplantation and in vivo bioluminescence imaging

The generation and characterization of the Tet-system transgenic lines of conditional expression of MYC have been described^3^. We subcutaneously transplanted 2 × 10^5^ PGK-RLuc- and Tet-FLuc-labeled MYC T-ALL cells (clone B11 or E12) in the flanks of 4- to 8-week-old NSG mice (*n* = 38). At specified times, or when the tumors reached a volume of 0.8 cm^3^, *MYC* was inactivated to induce tumor regression by treating mice with doxycycline (100 μg/ml i.p. injection on the first day and in drinking water (100 μg/ml) in the following days). The processes of tumor growth, regression and recurrence were monitored via bioluminescence imaging (BLI) of RLuc expression. MYC expression was monitored indirectly via BLI of Tet-FLuc expression. All BLI measurements were acquired using the Ami system (Spectral Instruments Imaging). For each mouse, RLuc substrate coelenterazine (50 µg in 150 µl saline) was injected intravenously and images were acquired immediately. Six hours later, FLuc substrate D-luciferin (33 mg/ml, 100 μl) was injected intraperitoneally and images were acquired after 10 min. BLI signals were quantified using Aura software (Spectral Instruments Imaging).

Compared to RLuc, FLuc has a longer emission wavelength and is therefore brighter and more efficient in penetrating light through tissue. To address this quantitatively, the minimum and range of signal for both FLuc and RLuc were calculated for each mouse and then averaged for each group of mice studied. The FLuc signal was normalized to the RLuc range using the following equation:

3 $$\frac{{F\!Luc}_{\text{1}}(t)-{F\!Luc}_{\text{min}}}{{F\!Luc}_{\text{range}}}=\frac{{F\!Luc}_{\text{2}}(t)-{R\!Luc}_{\text{min}}}{{R\!Luc}_{\text{range}}}$$

where *FLuc*_1_(*t*) is the raw FLuc signal at time *t*; *FLuc*_2_(*t*) is the normalized FLuc signal at time *t*; *FLuc*_min_ and *RLuc*_min_ are the minimum detected FLuc and RLuc signals, respectively; and *FLuc*_range_ and *RLuc*_range_ are the differences between the maximum and minimum FLuc and RLuc signals, respectively.

We performed in vivo RLuc and FLuc imaging on healthy mice (*n* = 10 age-matched mice without tumor xenografts) to determine the limit of detection of bioluminescence signal (1.58 × 10^5^ photon/s for RLuc; 1.64 × 10^5^ photon/s for FLuc). We defined any signal above 1.60 × 10^5^ photon/s to be imaging-detectable.

For the experiments with NK cell adoptive transfer, syngeneic NK cells were isolated from FVB/N mice (4–8 weeks old) by magnetic activated cell sorting with a NK cell selection kit (Miltenyi Biotec). Flow cytometry was performed to confirm the purity of the isolated NK cells. We intravenously injected 3 × 10^6^ PGK-FLuc labeled T-ALL cells (line 4188) into 4- to 8-week-old NSG mice (*n* = 16). Tumor engraftment was monitored by BLI. All mice were treated with doxycycline to inactivate *MYC* 7 days post-transplantation of tumor cells. Eight of 16 mice were subjected to adoptive transfer of NK cells (1 × 10^6^ week). In another experiment, one dose of NK cells (1 × 10^6^ cells) was adoptively transferred 3 days before tumor transplantation to evaluate the role of NK cells in tumor growth.

All animal experiments were approved by Stanford University's Administrative Panel on Laboratory Animal Care (APLAC) and were performed in accordance with institutional and national guidelines.

### Quantification of *MYC* mRNA using qPCR and Sanger sequencing of relapsed tumors

To measure the kinetics of *MYC* inactivation in vivo, mice were treated with doxycycline by intraperitoneal injection (100 μg/ml) when tumor volume reached 0.8 cm^3^. Samples were collected from the same tumor at 0, 1, 2, 4, 6, 8, and 10 h post-injection of doxycycline. To examine the levels of transgenic *MYC* in relapsed tumors, all tumor tissue was excised and collected at each experimental endpoint. Total mRNA was extracted from the tumor samples and relapsed tumors using an RNA extraction kit (RNeasy Mini Kit, Qiagen). Transgenic *MYC* mRNA expression was measured in triplicate using SYBR-based quantitative PCR and measurements were normalized to Ubiquitin C (UBC) mRNA levels. The primers used for transgenic *MYC* were: MYC-F, GGTCACACCCTTCTCCCTTC; MYC-R, AGCAGCTCGGTCACCATC.

To check whether the recurring tumors acquired mutations in the *tTA* gene, we excised all recurring tumors and extracted genomic DNA with a DNA extraction kit (NucleoSpin Tissue, Macherey–Nagel). The *tTA* region was amplified by PCR from genomic DNA using the Herculase II Fusion kit and directly sequenced in Stanford PAN facility (F-primer, CCTCAGTGGATGTTGCCTTT; R-primer, CCTGCACCTGAGGAGTGAAT).

### Western blotting and immunohistochemistry staining

Proteins were extracted using Bicine/CHAPS lysis buffer. Lysates were then separated via gel electrophoresis (Bio-Rad) and transferred to PVDF membranes using standard protocols. MYC and FLuc protein levels were detected using an anti-MYC (ab32072, Abcam)^51^ or anti-FLuc (ab21176, Abcam)^52^ antibody, respectively. The blot was imaged using a LI-COR scanner and analyzed by ImageJ.

Paraffin-embedded tumor sections were deparaffinized by successive incubation in xylene, 95% ethanol, 90% ethanol, and 70% ethanol, followed by PBS. Epitopes were unmasked by steaming in DAKO antigen retrieval solution for 45 min and then rinsed twice in PBS. The sections were blocked using DAKO blocking solution, and then immunostained overnight at 4 °C using primary antibodies (MYC, 1:150, Epitomics). Sections were then washed with PBS and incubated with biotinylated anti-rabbit IgG (1:300, Vector Labs) for 30 min at room temperature, then with the ABC kit (Vector Labs) for 30 min at room temperature. Sections were developed using 3,3′-diaminobenzidine (DAB, Vector Labs), counterstained with hematoxylin, and mounted with Permount. Images were obtained on a Philips Ultrafast Scanner.

### Intravital microscopic imaging (IVM)

Syngeneic NK cells were isolated from 4- to 6-week-old CAG-mCherry mice by magnetic activated cell sorting with a NK cell selection kit (Miltenyi Biotec). Adoptive transfer of 1 × 10^6^ NK cells to NSG mice was performed 3 days before tumor transplantation. For IVM imaging, dorsal skinfold chamber surgery was performed according to an established protocol^53^. Then 2 × 10^6^ GFP-labeled MYC T-ALL cells (line 4188) were transplanted subcutaneously in the chamber. After 4 days, tumor cells and NK cells were imaged simultaneously in vivo under a multiphoton microscope (Nikon A1 MP +) using a 10 × water immersion objective.

### Model fitting and parameter estimation

All model fits and simulations were performed using the simulation, analysis and modeling software SAAM II (The Epsilon Group). For the T-ALL experimental data studied here, the 3-compartment model used for curve fitting consisted of Eqs. 1d, 1e, 1f, 2a, 2b, and 2c, with *k*_on _= 0, and is depicted schematically in Supplementary Fig. 1a. Briefly, the rate of *MYC* inactivation (*k*_off_) was estimated by fitting a monoexponential decay function to *MYC* expression data measured by RT-PCR (Fig. 1c). Then, for each mouse, Eqs. 1d, 1e, 1f, 2a, 2b, and 2c were fitted to the total tumor BLI signal (e.g., RLuc total flux, shown in Supplementary Figs. 2, 4, 5, and 6) to estimate rate constants *k*_netgr,On_, *k*_netgr,Esc_, *k*_netdeath,Off_ and *k*_esc_. Best fitted curves were shown as black dashed lines (e.g., Fig. 2c for NSG mouse #18). Optimal parameter estimates for all mice in this study are provided explicitly in Supplementary Tables 1–10. Parameter estimates for each mouse were then used to predict (i.e., simulate) the number of cells in the MYC-On (blue curve), MYC-Off (red curve), and Esc (green curve) states.

The Akaike Information Criterion (AIC) was used to compare model fits, with the best fit indicated by the lowest AIC value^54^. All data generated and analyzed in this study are included in this published article (and its Supplementary Information files).

## Supplementary Information

Supplementary Information 1.

Supplementary Video 1.

Supplementary Video 2.
