# Supplementary material for: A mathematical model of tumor regression and recurrence after therapeutic oncogene inactivation
Source: Sci Rep. 2021 Jan 14;11:1341. doi: 10.1038/s41598-020-78947-2 (PMC7809285; doi:10.1038/s41598-020-78947-2)
Supplement: Supplementary file 1 — Supplementary Information 1. [file 41598_2020_78947_MOESM1_ESM.pdf]

# A Mathematical Model of Tumor Regression and Recurrence After Therapeutic Oncogene Inactivation

## SUPPLEMENTARY INFORMATION

Sharon S. Hori[1,2,3*†], Ling Tong[2,4*], Srividya Swaminathan[4,8], Mariola Liebersbach[4], Jingjing Wang[4,9], Sanjiv S. Gambhir[1,2,3,5,6], and Dean W. Felsher[2,4,7†]

1. Department of Radiology, Stanford University School of Medicine, Stanford, CA, USA.
2. Molecular Imaging Program at Stanford, Stanford University School of Medicine, Stanford, CA, USA.
3. Canary Center at Stanford for Cancer Early Detection, Stanford University School of Medicine, Palo Alto, CA, USA.
4. Division of Oncology, Department of Medicine, Stanford University School of Medicine, Stanford, CA, USA.
5. Department of Bioengineering, Stanford University School of Medicine, Stanford, CA, USA.
6. Department of Materials Science and Engineering, Stanford University, Stanford, CA, USA.
7. Department of Pathology, Stanford University School of Medicine, Stanford, CA, USA.
8. Present Address: Department of Systems Biology, Beckman Research Institute of the City of Hope, Monrovia, CA, USA.
9. Present Address: Department of Oncology, The Second Xiangya Hospital of Central South University, Changsha, People's Republic of China.

*Co-first authors, †Co-senior authors.

Address correspondence to shori@stanford.edu and dfelsher@stanford.edu.

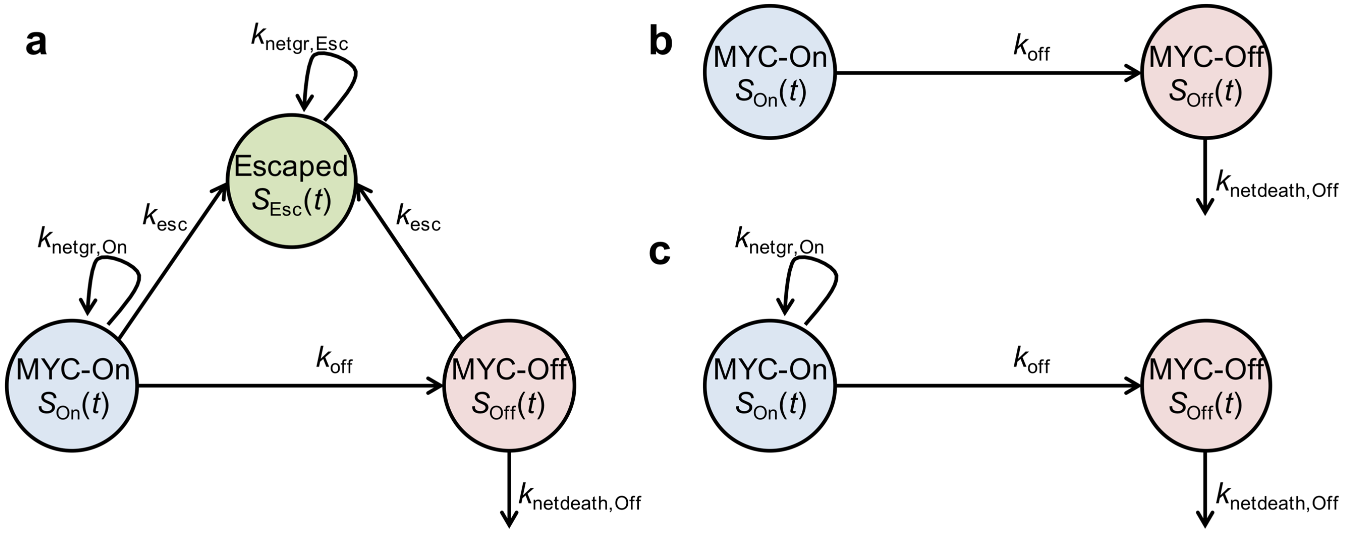


**Supplementary Fig. 1.** Submodels of the 3-compartment model. **(a)** The simplified 3-compartment model with net-growth and net-death rates. **(b)** A 2-compartment submodel without net-growth of the MYC-On compartment. **(c)** A 2-compartment submodel including net-growth of the MYC-On compartment.

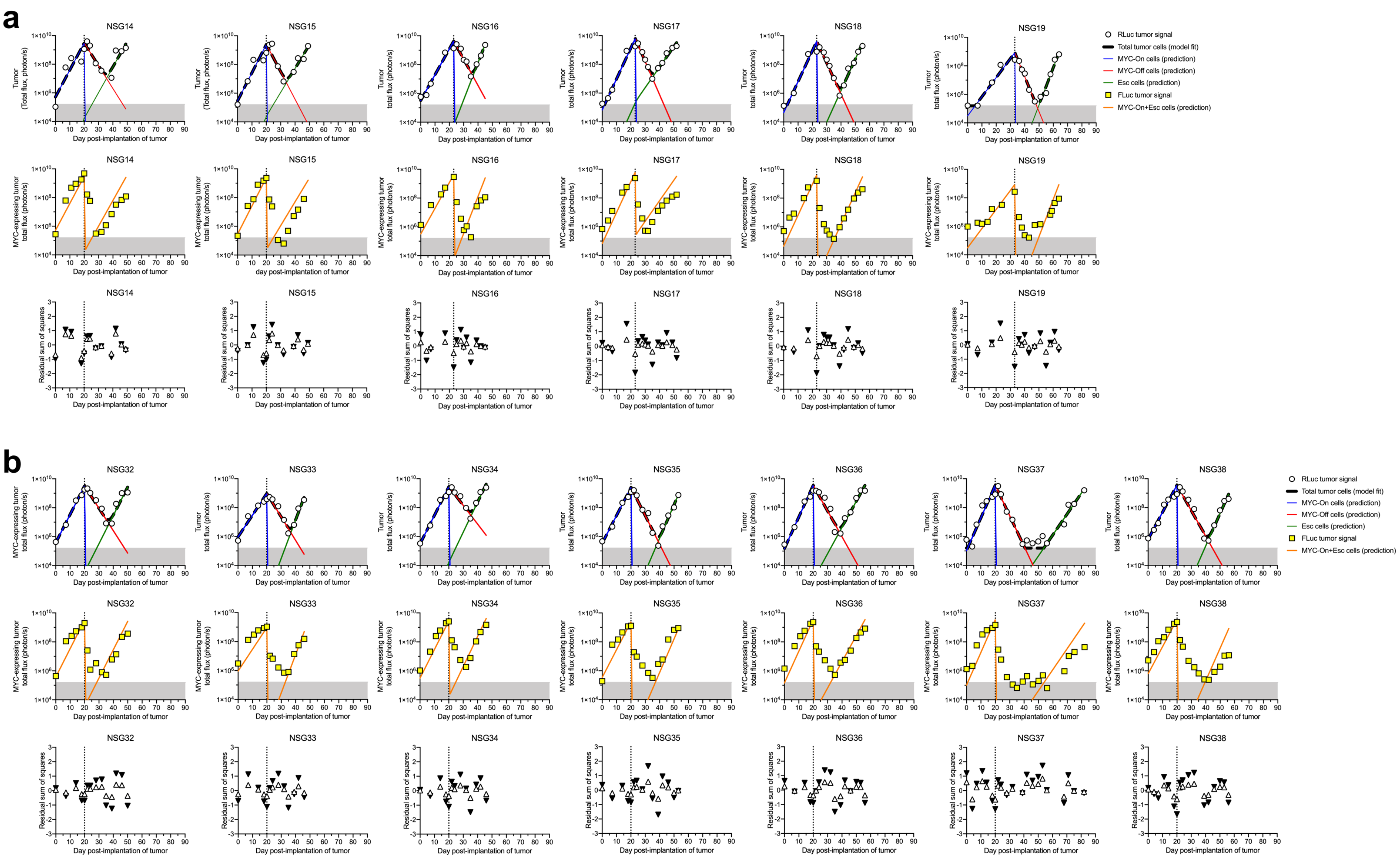


**Supplementary Fig. 2.** Mice implanted with subcutaneous xenografts of $2{\times}10^5$ 4188-PGK-RLuc/Tet-FLuc cells based on a single clonal population **(a)** B11 or **(b)** E12, with doxycycline (Dox) administration (dotted black vertical line) at Day 20, 23 or 33. Top rows show the fitting of the simplified 3-compartment model (dashed black line) to RLuc signal (white circles), superimposed with the predicted population of MYC-On cells (blue line), MYC-Off cells (red line), and Escaped cells (green line). Shaded grey region (below $1.60{\times}10^5$ photon/s) indicates imaging signal is below the limit of detection. Middle rows show the model-predicted MYC-expressing tumor cell population (orange line) superimposed

with Tet-FLuc signal (yellow squares). Bottom rows show the unweighted (white triangles) or weighted (black triangles) residual sum of squares indicating the deviation between experimental data and predicted value at each time point.

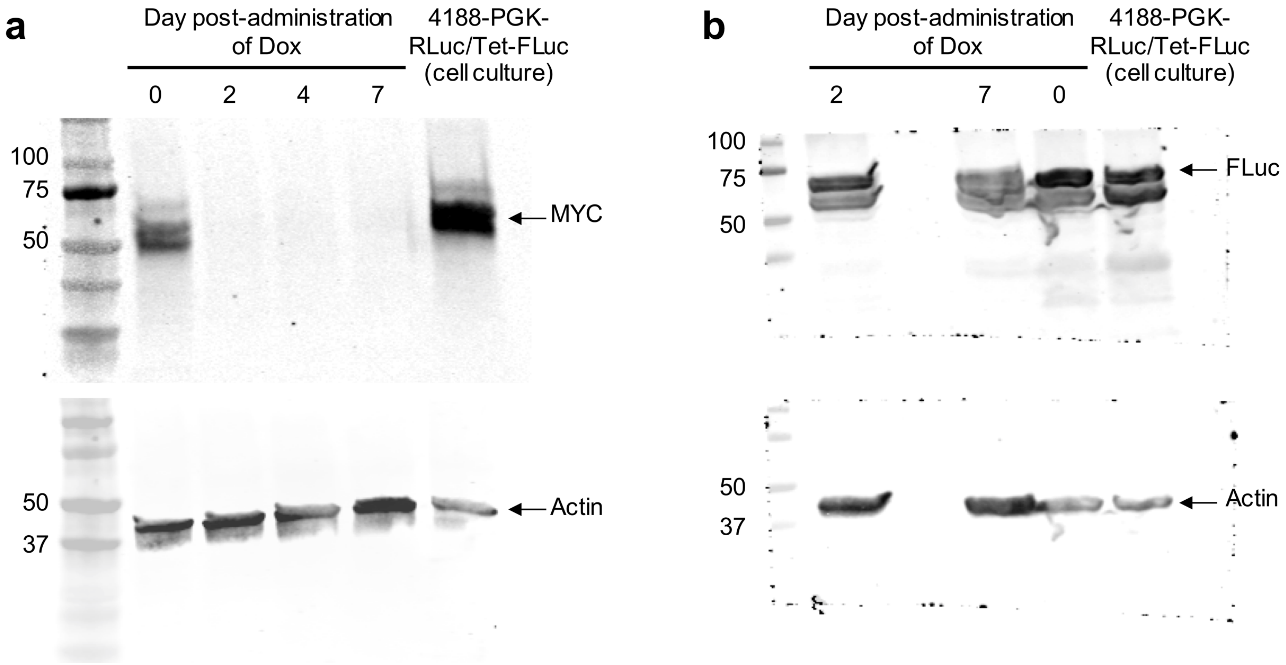


**Supplementary Fig. 3.** Western blots of **(a)** MYC and **(b)** Firefly luciferase (FLuc) expression in the tumors collected immediately before (day 0), or days 2 or 7 after doxycycline (Dox) was administered to inactivate MYC. Cultured 4188-PGK-RLuc/Tet-FLuc cells (Clone B11) were used as a positive control. MYC, FLuc and actin bands are indicated by arrows. Bands for days 0, 2, and 7 are shown in **Fig. 2g**.

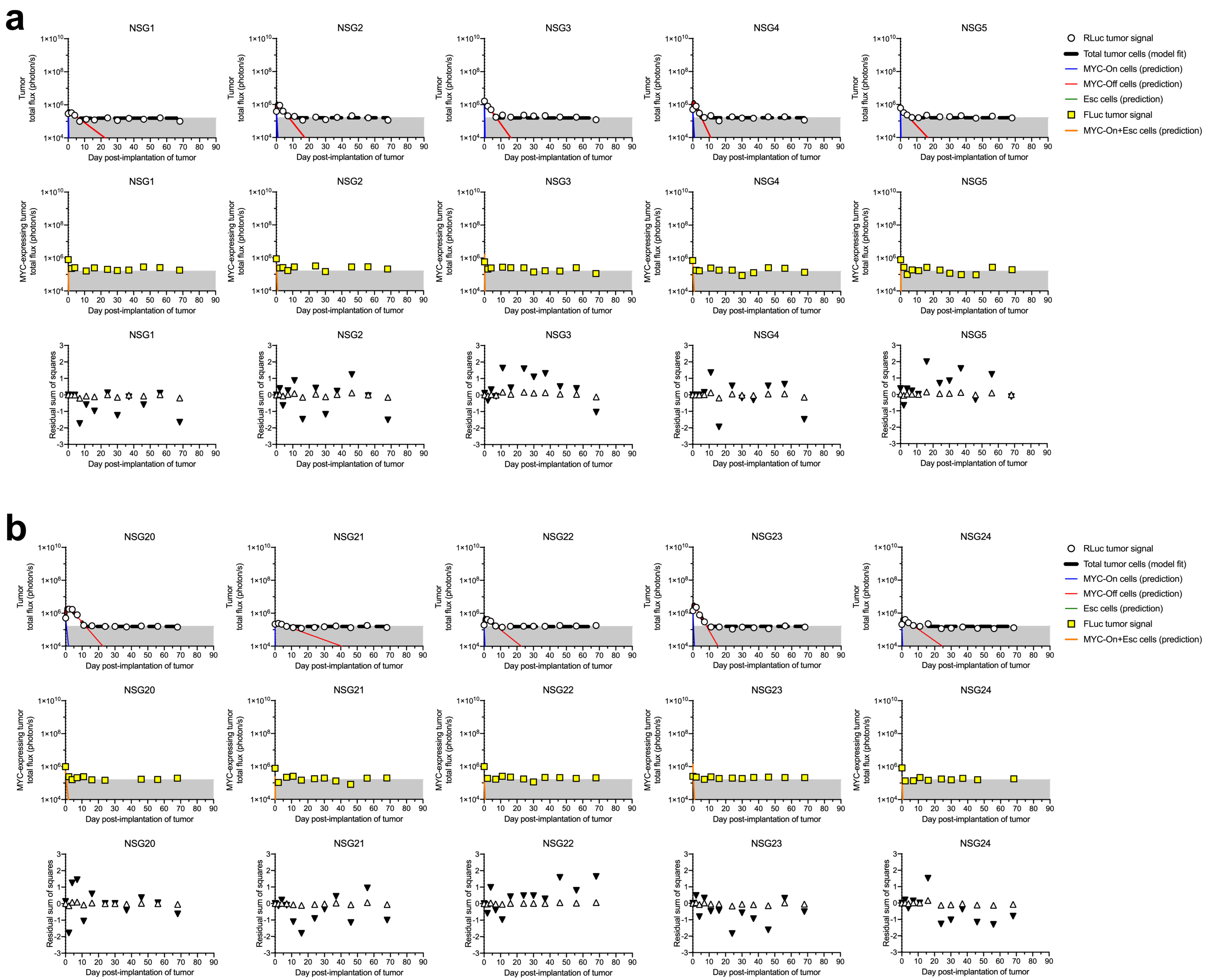


**Supplementary Figure 4**. Mice implanted with subcutaneous xenografts of $2\times10^5$ 4188-PGK-RLuc-Tet-FLuc cells based on a single clonal population **(a)** B11 or **(b)** E12, with doxycycline (Dox) administration (dotted black vertical line) at Day 0. Top rows show the fitting of the

simplified 3-compartment model (dashed black line) to RLuc signal (white circles), superimposed with the predicted population of MYC-On cells (blue line), MYC-Off cells (red line), and Escaped cells (green line). Shaded grey region (below $1.60\times10^5$ photon/s) indicates imaging signal is below the limit of detection. Middle rows show the model-predicted MYC-expressing tumor cell population (orange line) superimposed with Tet-FLuc signal (yellow squares). Bottom rows show the unweighted (white triangles) or weighted (black triangles) residual sum of squares indicating the deviation between experimental data and predicted value at each time point.

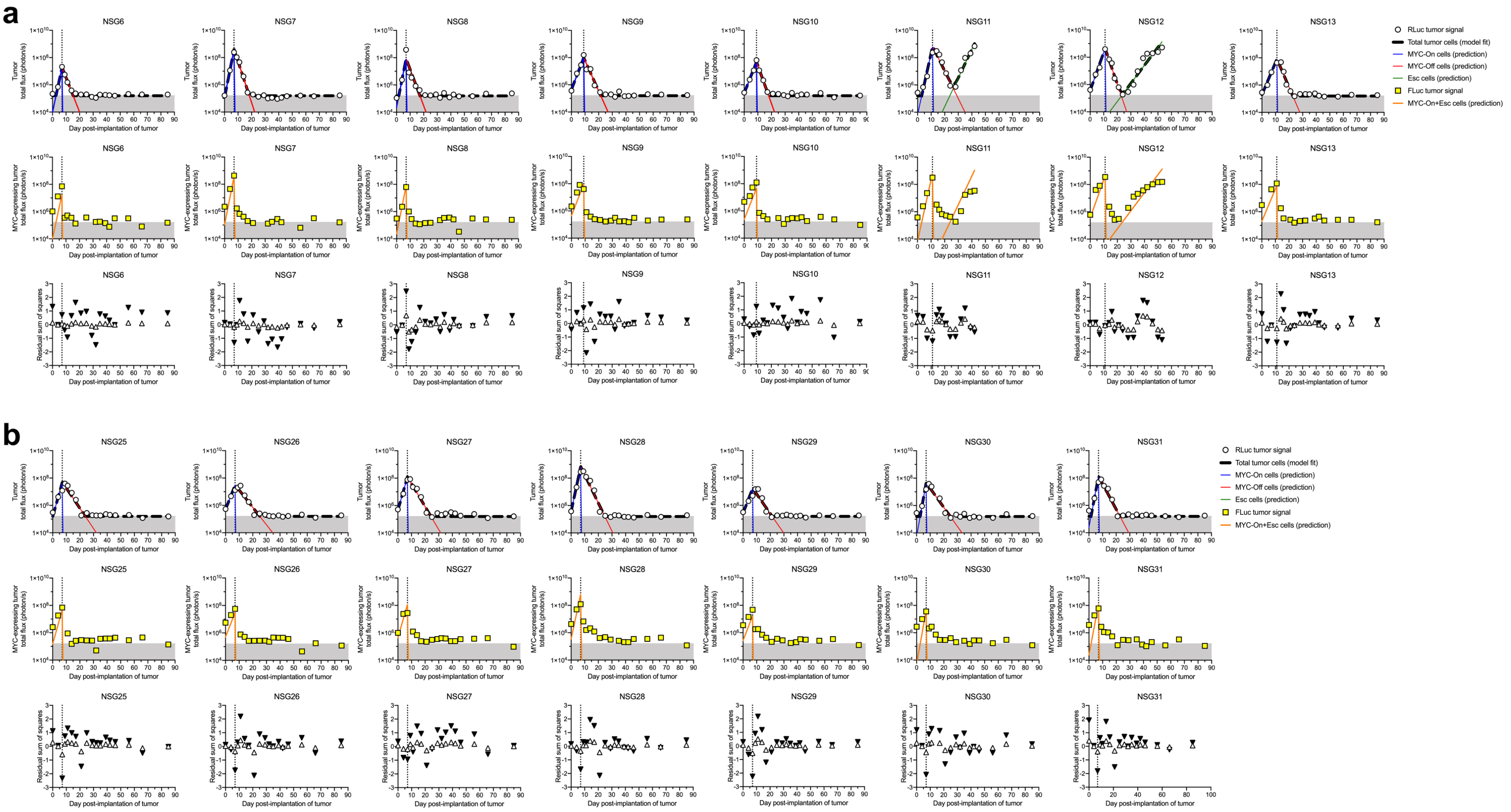


**Supplementary Figure 5**. Mice implanted with subcutaneous xenografts of $2\times10^5$ 4188-PGK-RLuc-Tet-FLuc cells based on a single clonal population **(a)** B11 or **(b)** E12, with doxycycline (Dox) administration (dotted black vertical line) at indicated times (Day 7, 9, or 11). Top rows show the fitting of the simplified 3-compartment model (dashed black line) to RLuc signal (white circles), superimposed with the predicted population of MYC-On cells (blue line), MYC-Off cells (red line), and Escaped cells (green line). Shaded grey region (below $1.60\times10^5$ photon/s) indicates imaging signal is below the limit of detection. Middle rows show the model-predicted MYC-expressing tumor cell population (orange line) superimposed with Tet-FLuc signal (yellow squares). Bottom rows show the unweighted (white triangles) or weighted (black triangles) residual sum of squares indicating the deviation between experimental data and predicted value at each time point.

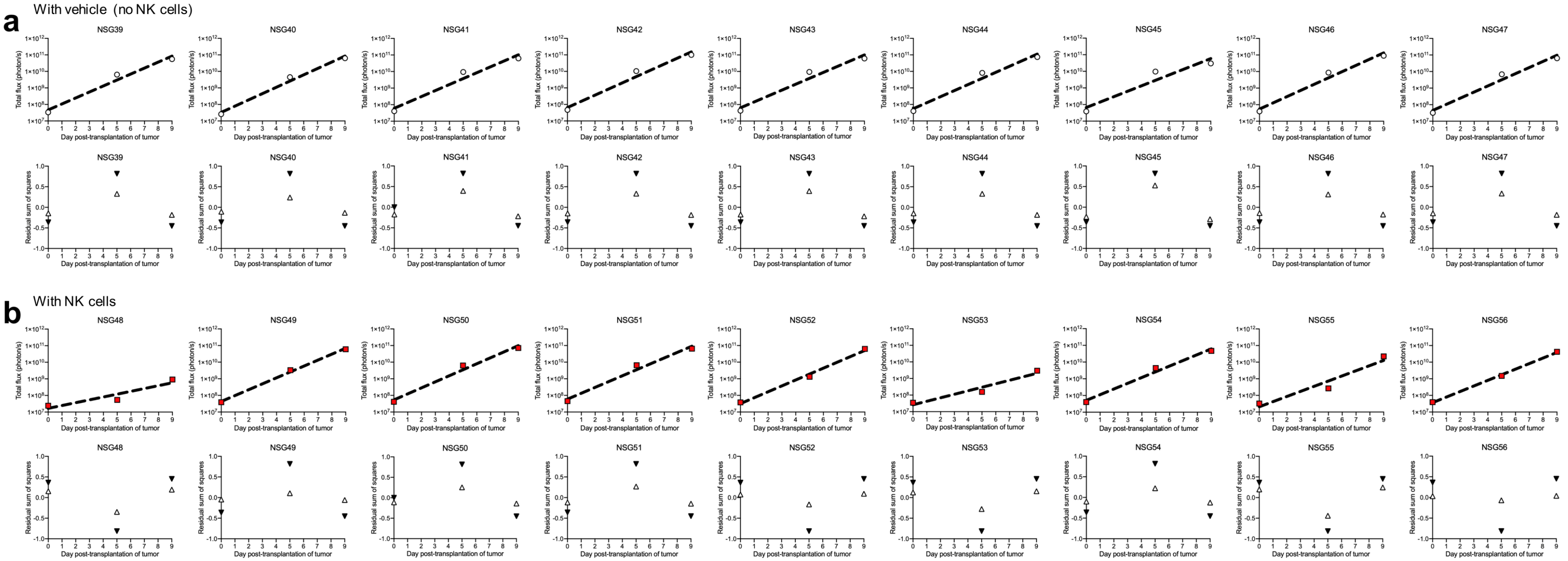


**Supplementary Figure 6**. Natural killer (NK) cells delay tumor growth. NSG mice received either **(a)** a saline injection or **(b)** $1\times10^6$ NK cells via tail vein three days before intravenous transplantation of $3\times10^6$ 4188-PGK-FLuc cells. Tumor growth was then monitored by firefly luciferase (FLuc) imaging.

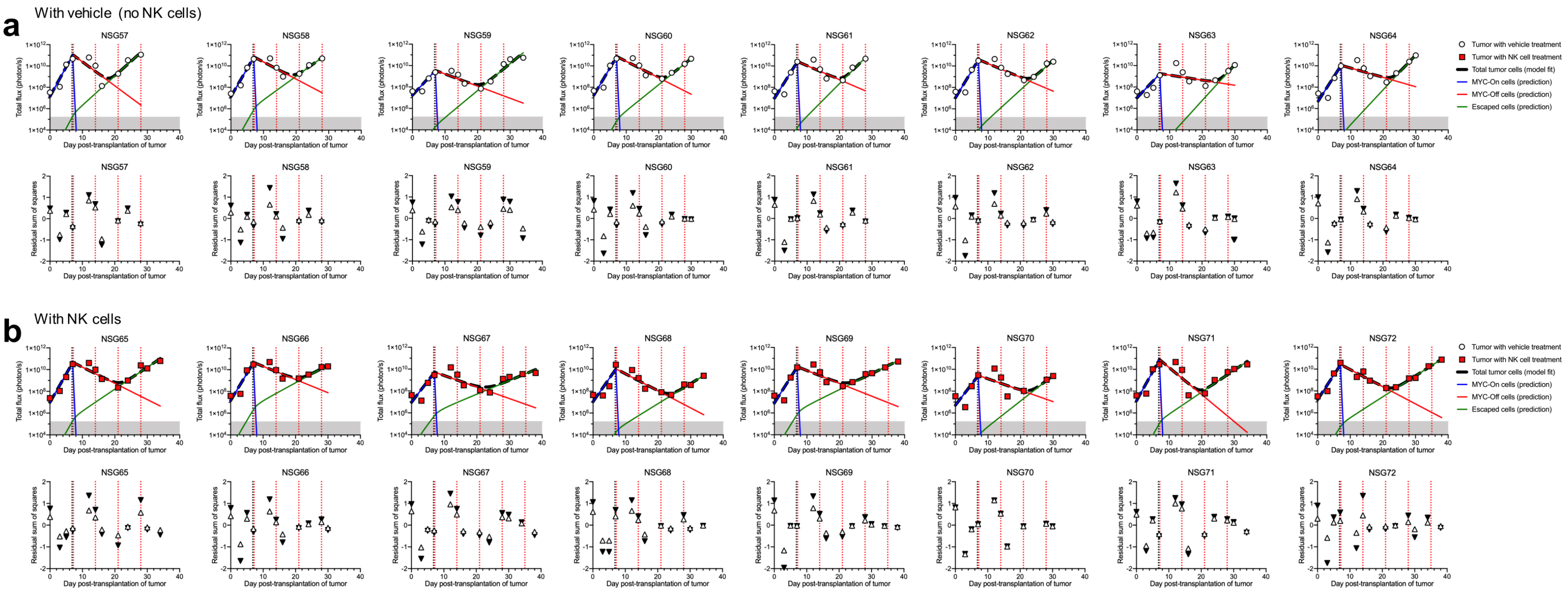


**Supplementary Figure 7**. Natural killer (NK) cells delay tumor recurrence. NSG mice (*n*=16) were intravenously injected with $3\times10^6$ 4188-PGK-FLuc cells and monitored by firefly luciferase (FLuc) imaging. Doxycycline (Dox) was administered 7 days later to inactivate MYC oncogene. On the day of Dox administration (black dotted vertical line) and weekly thereafter (red dotted vertical lines), mice received either **(a)** a saline injection or **(b)** adoptive transfer of $1\times10^6$ NK cells. Top rows show the fitting of the simplified 3-compartment model (dashed black line) to FLuc signal (white circles), superimposed with the predicted population of MYC-On cells (blue line), MYC-Off cells (red line), and Escaped cells (green line). Shaded grey region (below $1.60\times10^5$ photon/s) indicates imaging signal is below the limit of detection. Bottom rows show the unweighted (white triangles) or weighted (black triangles) residual sum of squares indicating the deviation between experimental data and predicted value at each time point.

## Supplementary Tables

The percent coefficient of variation (%CV), also called the relative standard deviation, is defined to be the standard deviation of the parameter estimate divided by the mean of the parameter estimate. The %CV provides a measure of the uncertainty of a parameter estimate, with a higher %CV corresponding to a greater uncertainty.

**Supplementary Table 1**. Clone B11
Dox administered on Day 0 post-implantation of $2{\times}10^5$ cells

| Parameter | Units | NSG1 | NSG2 | NSG3 | NSG4 | NSG5 |
|---|---|---|---|---|---|---|
| | | Value (%CV) | Value (%CV) | Value (%CV) | Value (%CV) | Value (%CV) |
| $N_{On,0}$ | photon/s | $2.95{\times}10^5$ (26) | $3.84{\times}10^5$ (23) | $1.62{\times}10^6$ (20) | $4.98{\times}10^5$ (22) | $5.72{\times}10^5$ (16) |
| $k_{esc}$ | day$^{-1}$ | 0 (fixed) | 0 (fixed) | 0 (fixed) | 0 (fixed) | 0 (fixed) |
| $k_{netdeath,Off}$ | day$^{-1}$ | $1.71{\times}10^{-1}$ (108) | $2.89{\times}10^{-1}$ (22) | $3.18{\times}10^{-1}$ (15) | $4.88{\times}10^{-1}$ (33) | $2.45{\times}10^{-1}$ (26) |
| $k_{netgr,On}$ | day$^{-1}$ | 5.73 (111) | $1.15{\times}10^1$ (13) | 0 (fixed) | $1.17{\times}10^1$ (17) | 0 (fixed) |
| $k_{netgr,Esc}$ | day$^{-1}$ | 0 (fixed) | 0 (fixed) | 0 (fixed) | 0 (fixed) | 0 (fixed) |

**Supplementary Table 2.** Clone B11
Dox administered on Day 7 post-implantation of $2{\times}10^5$ cells

| Parameter | Units | NSG6 | NSG7 | NSG8 |
|---|---|---|---|---|
| | | Value (%CV) | Value (%CV) | Value (%CV) |
| $N_{On,0}$ | photon/s | $9.51{\times}10^3$ (62) | $1.05{\times}10^5$ (79) | $2.30{\times}10^4$ (162) |
| $k_{esc}$ | day$^{-1}$ | 0 (fixed) | 0 (fixed) | 0 (fixed) |
| $k_{netdeath,Off}$ | day$^{-1}$ | $5.83{\times}10^{-1}$ (8) | $6.97{\times}10^{-1}$ (6) | $6.15{\times}10^{-1}$ (13) |
| $k_{netgr,On}$ | day$^{-1}$ | 1.08 (9) | 1.17 (11) | 1.16 (23) |
| $k_{netgr,Esc}$ | day$^{-1}$ | 0 (fixed) | 0 (fixed) | 0 (fixed) |

**Supplementary Table 3.** Clone B11
Dox administered on Day 9 post-implantation of $2{\times}10^5$ cells

| Parameter | Units | NSG9 | NSG10 |
|---|---|---|---|
| | | Value (%CV) | Value (%CV) |
| $N_{On,0}$ | photon/s | $4.44{\times}10^5$ (39) | $2.43{\times}10^5$ (23) |
| $k_{esc}$ | day$^{-1}$ | 0 (fixed) | 0 (fixed) |
| $k_{netdeath,Off}$ | day$^{-1}$ | $5.14{\times}10^{-1}$ (8) | $6.45{\times}10^{-1}$ (6) |
| $k_{netgr,On}$ | day$^{-1}$ | $5.95{\times}10^{-1}$ (10) | $5.85{\times}10^{-1}$ (6) |
| $k_{netgr,Esc}$ | day$^{-1}$ | 0 (fixed) | 0 (fixed) |

**Supplementary Table 4.** Clone B11
Dox administered on Day 11 post-implantation of $2{\times}10^5$ cells

| Parameter | Units | NSG11 | NSG12 | NSG13 |
|---|---|---|---|---|
| | | Value (%CV) | Value (%CV) | Value (%CV) |
| $N_{On,0}$ | photon/s | $6.66{\times}10^3$ (121) | $3.58{\times}10^5$ (71) | $1.98{\times}10^5$ (38) |
| $k_{esc}$ | day$^{-1}$ | $1.79{\times}10^{-7}$ (208) | $2.12{\times}10^{-6}$ (112) | 0 (fixed) |
| $k_{netdeath,Off}$ | day$^{-1}$ | $4.66{\times}10^{-1}$ (17) | $6.73{\times}10^{-1}$ (16) | $5.33{\times}10^{-1}$ (11) |
| $k_{netgr,On}$ | day$^{-1}$ | 1.04 (13) | $6.45{\times}10^{-1}$ (15) | $5.44{\times}10^{-1}$ (9) |
| $k_{netgr,Esc}$ | day$^{-1}$ | $4.83{\times}10^{-1}$ (17) | $3.05{\times}10^{-1}$ (11) | 0 (fixed) |

**Supplementary Table 5.** Clone B11
Dox administered on Day 20 post-implantation of 2×10$^5$ cells

| Parameter | Units | NSG14 | NSG15 |
|---|---|---|---|
| | | Value (%CV) | Value (%CV) |
| $N_{On,0}$ | photon/s | 5.11×10$^5$ (130) | 2.88×10$^5$ (105) |
| $k_{esc}$ | day$^{-1}$ | 3.18×10$^{-7}$ (721) | 8.38×10$^{-7}$ (534) |
| $k_{netdeath,Off}$ | day$^{-1}$ | 3.67×10$^{-1}$ (43) | 4.41×10$^{-1}$ (31) |
| $k_{netgr,On}$ | day$^{-1}$ | 4.33×10$^{-1}$ (20) | 4.52×10$^{-1}$ (16) |
| $k_{netgr,Esc}$ | day$^{-1}$ | 4.12×10$^{-1}$ (53) | 3.83×10$^{-1}$ (45) |

**Supplementary Table 6.** Clone B11
Dox administered on Day 23 post-implantation of 2×10$^5$ cells

| Parameter | Units | NSG16 | NSG17 | NSG18 |
|---|---|---|---|---|
| | | Value (%CV) | Value (%CV) | Value (%CV) |
| $N_{On,0}$ | photon/s | 3.20×10$^5$ (52) | 6.82×10$^4$ (64) | 4.57×10$^4$ (123) |
| $k_{esc}$ | day$^{-1}$ | 3.95×10$^{-9}$ (678) | 5.52×10$^{-6}$ (162) | 2.54×10$^{-9}$ (315) |
| $k_{netdeath,Off}$ | day$^{-1}$ | 4.19×10$^{-1}$ (19) | 5.29×10$^{-1}$ (16) | 4.99×10$^{-1}$ (15) |
| $k_{netgr,On}$ | day$^{-1}$ | 4.12×10$^{-1}$ (8) | 4.96×10$^{-1}$ (8) | 4.94×10$^{-1}$ (13) |
| $k_{netgr,Esc}$ | day$^{-1}$ | 5.92×10$^{-1}$ (31) | 3.28×10$^{-1}$ (18) | 5.06×10$^{-1}$ (16) |

**Supplementary Table 7.** Clone B11
Dox administered on Day 33 post-implantation of 2×10$^5$ cells

| Parameter | Units | NSG19 |
|---|---|---|
| | | Value (%CV) |
| $N_{On,0}$ | photon/s | 3.11×10$^4$ (83) |
| $k_{esc}$ | day$^{-1}$ | 1.41×10$^{-13}$ (432) |
| $k_{netdeath,Off}$ | day$^{-1}$ | 5.64×10$^{-1}$ (12) |
| $k_{netgr,On}$ | day$^{-1}$ | 3.09×10$^{-1}$ (11) |
| $k_{netgr,Esc}$ | day$^{-1}$ | 6.03×10$^{-1}$ (13) |

**Supplementary Table 8.** Clone E12
Dox administered on Day 0 post-implantation of 2×10$^5$ cells

| Parameter | Units | NSG20 | NSG21 | NSG22 | NSG23 | NSG24 |
|---|---|---|---|---|---|---|
| | | Value (%CV) | Value (%CV) | Value (%CV) | Value (%CV) | Value (%CV) |
| $N_{On,0}$ | photon/s | 4.98×10$^5$ (16) | 2.41×10$^5$ (12) | 1.95×10$^5$ (9) | 1.39×10$^6$ (21) | 2.07×10$^5$ (24) |
| $k_{esc}$ | day$^{-1}$ | 0 (fixed) | 0 (fixed) | 0 (fixed) | 0 (fixed) | 0 (fixed) |
| $k_{netdeath,Off}$ | day$^{-1}$ | 2.66×10$^{-1}$ (9) | 4.91×10$^{-2}$ (59) | 1.83×10$^{-1}$ (14) | 4.01×10$^{-1}$ (14) | 1.62×10$^{-2}$ (42) |
| $k_{netgr,On}$ | day$^{-1}$ | 1.38×10$^1$ (3) | 0 (fixed) | 1.09×10$^1$ (7) | 1.07×10$^1$ (15) | 9.72 (25) |
| $k_{netgr,Esc}$ | day$^{-1}$ | 0 (fixed) | 0 (fixed) | 0 (fixed) | 0 (fixed) | 0 (fixed) |

**Supplementary Table 9.** Clone E12
Dox administered on Day 7 post-implantation of 2×$10^5$ cells

| Parameter | Units | NSG25 | NSG26 | NSG27 | NSG28 | NSG29 | NSG30 | NSG31 |
|---|---|---|---|---|---|---|---|---|
| | | Value (%CV) | Value (%CV) | Value (%CV) | Value (%CV) | Value (%CV) | Value (%CV) | Value (%CV) |
| $N_{On,0}$ | photon/s | 1.49×$10^5$ (144) | 5.07×$10^5$ (44) | 4.32×$10^5$ (44) | 3.47×$10^5$ (44) | 2.69×$10^5$ (50) | 5.32×$10^3$ (121) | 2.46×$10^4$ (123) |
| $k_{esc}$ | day$^{-1}$ | 0 (fixed) | 0 (fixed) | 0 (fixed) | 0 (fixed) | 0 (fixed) | 0 (fixed) | 0 (fixed) |
| $k_{netdeath,Off}$ | day$^{-1}$ | 3.40×$10^{-1}$ (15) | 2.91×$10^{-1}$ (10) | 3.86×$10^{-1}$ (10) | 4.72×$10^{-1}$ (8) | 3.30×$10^{-1}$ (14) | 3.20×$10^{-1}$ (13) | 4.29×$10^{-1}$ (10) |
| $k_{netgr,On}$ | day$^{-1}$ | 8.21×$10^{-1}$ (28) | 5.82×$10^{-1}$ (13) | 8.01×$10^{-1}$ (10) | 1.07 (7) | 5.93×$10^1$ (15) | 1.28 (15) | 1.22 (16) |
| $k_{netgr,Esc}$ | day$^{-1}$ | 0 (fixed) | 0 (fixed) | 0 (fixed) | 0 (fixed) | 0 (fixed) | 0 (fixed) | 0 (fixed) |

**Supplementary Table 10.** Clone E12
Dox administered on Day 20 post-implantation of 2×$10^5$ cells

| Parameter | Units | NSG32 | NSG33 | NSG34 | NSG35 | NSG36 | NSG37 | NSG38 |
|---|---|---|---|---|---|---|---|---|
| | | Value (%CV) | Value (%CV) | Value (%CV) | Value (%CV) | Value (%CV) | Value (%CV) | Value (%CV) |
| $N_{On,0}$ | photon/s | 3.74×$10^5$ (67) | 9.23×$10^5$ (66) | 3.13×$10^5$ (64) | 3.47×$10^5$ (67) | 1.47×$10^5$ (139) | 1.03×$10^5$ (103) | 6.46×$10^5$ (58) |
| $k_{esc}$ | day$^{-1}$ | 5.11×$10^{-8}$ (364) | 9.13×$10^{-11}$ (660) | 2.18×$10^{-7}$ (481) | 1.31×$10^{-10}$ (369) | 2.63×$10^{-8}$ (270) | 8.29×$10^{-11}$ (318) | 3.41×$10^{-11}$ (441) |
| $k_{netdeath,Off}$ | day$^{-1}$ | 3.57×$10^{-1}$ (17) | 3.73×$10^{-1}$ (16) | 2.99×$10^{-1}$ (23) | 4.55×$10^{-1}$ (11) | 4.10×$10^{-1}$ (16) | 4.86×$10^{-1}$ (13) | 4.07×$10^{-1}$ (12) |
| $k_{netgr,On}$ | day$^{-1}$ | 4.52×$10^{-1}$ (10) | 3.46×$10^{-1}$ (12) | 4.54×$10^{-1}$ (9) | 4.43×$10^{-1}$ (10) | 5.03×$10^{-1}$ (16) | 5.21×$10^{-1}$ (13) | 4.30×$10^{-1}$ (9) |
| $k_{netgr,Esc}$ | day$^{-1}$ | 4.52×$10^{-1}$ (23) | 6.11×$10^{-1}$ (28) | 4.79×$10^{-1}$ (31) | 5.33×$10^{-1}$ (17) | 4.18×$10^{-1}$ (18) | 3.35×$10^{-1}$ (17) | 5.18×$10^{-1}$ (20) |
